# Dynamic profiles of lncRNAs reveal a functional natural antisense RNA that regulates the development of *Schistosoma japonicum*

**DOI:** 10.1371/journal.ppat.1011949

**Published:** 2024-01-29

**Authors:** Shaoyun Cheng, Yanmin You, Xiaoling Wang, Cun Yi, Wei Zhang, Yuxiang Xie, Lei Xiu, Fang Luo, Yan Lu, Jipeng Wang, Wei Hu

**Affiliations:** 1 State Key Laboratory of Genetic Engineering, Ministry of Education Key Laboratory of Contemporary Anthropology, School of Life Sciences, Fudan University, Shanghai, China; 2 National Institute of Parasitic Diseases, Chinese Center for Disease Control and Prevention (Chinese Center for Tropical Diseases Research), NHC Key Laboratory of Parasite and Vector Biology, WHO Collaborating Center for Tropical Diseases, National Center for International Research on Tropical Diseases, Shanghai, China; 3 College of Life Sciences, Inner Mongolia University, Hohhot, Inner Mongolia Autonomous Region, China; 4 Department of Infectious Diseases, Huashan Hospital, Fudan University, Shanghai, China; Rush University Medical Center, UNITED STATES

## Abstract

Schistosomes are flatworm parasites that undergo a complex life cycle involving two hosts. The regulation of the parasite’s developmental processes relies on both coding RNAs and non-coding RNAs. However, the roles of non-coding RNAs, including long non-coding RNAs (lncRNAs) in schistosomes remain largely unexplored. Here we conduct advanced RNA sequencing on male and female *S*. *japonicum* during their pairing and reproductive development, resulting in the identification of nearly 8,000 lncRNAs. This extensive dataset enables us to construct a comprehensive co-expression network of lncRNAs and mRNAs, shedding light on their interactions during the crucial reproductive stages within the mammalian host. Importantly, we have also revealed a specific lncRNA, *LNC3385*, which appears to play a critical role in the survival and reproduction of the parasite. These findings not only enhance our understanding of the dynamic nature of lncRNAs during the reproductive phase of schistosomes but also highlight *LNC3385* as a potential therapeutic target for combating schistosomiasis.

## Author summary

Schistosomiasis, a disease that affects millions, has only one drug available treatment, making it essential for us to uncover other potential therapeutic avenues. In our quest to understand the disease better, we delved into the role of long non-coding RNAs (lncRNAs) during the parasite’s reproductive phase, a crucial period for the disease’s spread. Using advanced RNA sequencing, we identified nearly 8,000 lncRNAs during the parasite’s reproductive development. Most intriguingly, we identified one specific lncRNA, named *LNC3385*, that appears vital for the parasite’s survival and reproduction. Our study, therefore, not only provides a deeper understanding of the molecular dynamics during the reproductive phase of the parasite but also highlights a potential new target, *LNC3385*, for therapeutic strategies against schistosomiasis.

## Introduction

Schistosomiasis is a neglected tropical disease caused by blood flukes of the genus Schistosoma, which infects over 230 million people worldwide and results in an estimated ~280,000 deaths annually [1]. The clinical symptoms and transmission of this disease are due to the large number of eggs produced by mature female worms, the reproductive maturation of which depends on continuous pairing with male parasites [2–4]. Only one drug (praziquantel) is currently available to effectively treat schistosomiasis. Therefore, a comprehensive understanding of the reproductive development process of schistosomes could provide new insights for disease control and drug development.

As the only platyhelminths that have evolved separate sexes, schistosomes possess a unique and complex reproductive development process involving interactions between male and female worms and intricate molecular regulatory events [2,5]. Previous studies have demonstrated that dramatic expression changes occurred in protein-coding genes as well as non-coding genes, such as miRNA, in both genders along with their pairing and sexual development [6,7], suggesting a potential role of these non-coding genes in schistosome development and reproduction. The non-coding RNAs including miRNAs, are widespread in the genome of schistosomes [8–11]. Indeed, serval miRNAs have been characterized in the regulation of ovarian development in *S*. *japonicum* [12]. However, the functions of lncRNAs in schistosomes are poorly understood.

LncRNAs are non-coding RNAs with a length longer than 200 nucleotides that play a role in transcriptional, post-transcriptional, and other modes of gene regulation by interacting with DNA, RNA, proteins, or chromatin [13,14]. In general, they could regulate the gene expression in two ways, one is affecting the transcription of their adjacent genes *in cis*, and the other is regulating mRNA splicing, turnover, translation, and signaling pathways *in trans* [15]. In many species, lncRNAs have been found to play critical roles in regulating development and reproduction [16], including myoblast and cardiovascular differentiation [17,18], sex determination [19], gonadogenesis [20], sex hormone response [21], meiosis [22] and spermatogenesis [23]. In parasites, lncRNAs have been recognized as crucial regulators in development, sex differentiation and the host-parasite interaction according to the studies on malaria parasites [24,25] and *Trypanosoma brucei* [26]. Until recently, the lncRNAs in schistosomes have been identified from the genome and RNA-seq data, revealing over 10,000 lncRNAs [27], which is almost identical to the number of the protein-coding RNAs. As expected, these lncRNAs displayed dynamic expression profiles at different life stages of *Schistosoma mansoni* including egg, miracidia, cercariae, schistosomula (24h), adult male and female [27–31]. Morales-Vicente *et al*. revealed the tissue-specific expression patterns of lncRNAs by merging them with the single-cell RNA-seq atlas of *S*. *mansoni* [32]. Silveira *et al*. obtained pairing-dependent lncRNAs by re-analyzing the public RNA-Seq data of the paired and unpaired adult male and female worms as well as their gonads, identifying three lncRNAs essential for cell proliferation in female vitellaria [33]. These findings indicate that the expression patterns of lncRNAs are highly associated with the life stage, sex, and cell type of this parasite, highlighting the potential role of lncRNAs in regulating schistosome growth and development. However, the detailed expression profiles of lncRNAs and their roles during the male-female interaction and their reproductive development are lacking.

According to our pervious study, the male and female *S*. *japonicum* start pairing around 16 dpi and reach to sexually mature around 26 dpi in mice model [7]. Therefore, in this study, we focused on four stages throughout the male-female pairing and reproductive development, which were 14 dpi (pre-pairing stage), 18 dpi (post-pairing stage), 22 dpi (pre-mature stage) and 26 dpi (mature stage). We identified 7,975 lncRNAs and provided their dynamic expression profiles in both sexes of *S*. *japonicum* across these four stages by performing strand-specific RNA-seq (ssRNA-seq). We then constructed a comprehensive lncRNA-mRNA co-expression network throughout this process and clustered lncRNAs into modules representing different expression patterns. Through these upregulated lncRNAs during pairing, we identified a NAT-lncRNA *LNC3385* required for the development and reproduction of *S*. *japonicum*. This work highlights the importance of lncRNA in schistosome biology.

## Results

### Identification and characterization of lncRNAs in *S*. *japonicum* from 14 to 26dpi

**Fig 1:**
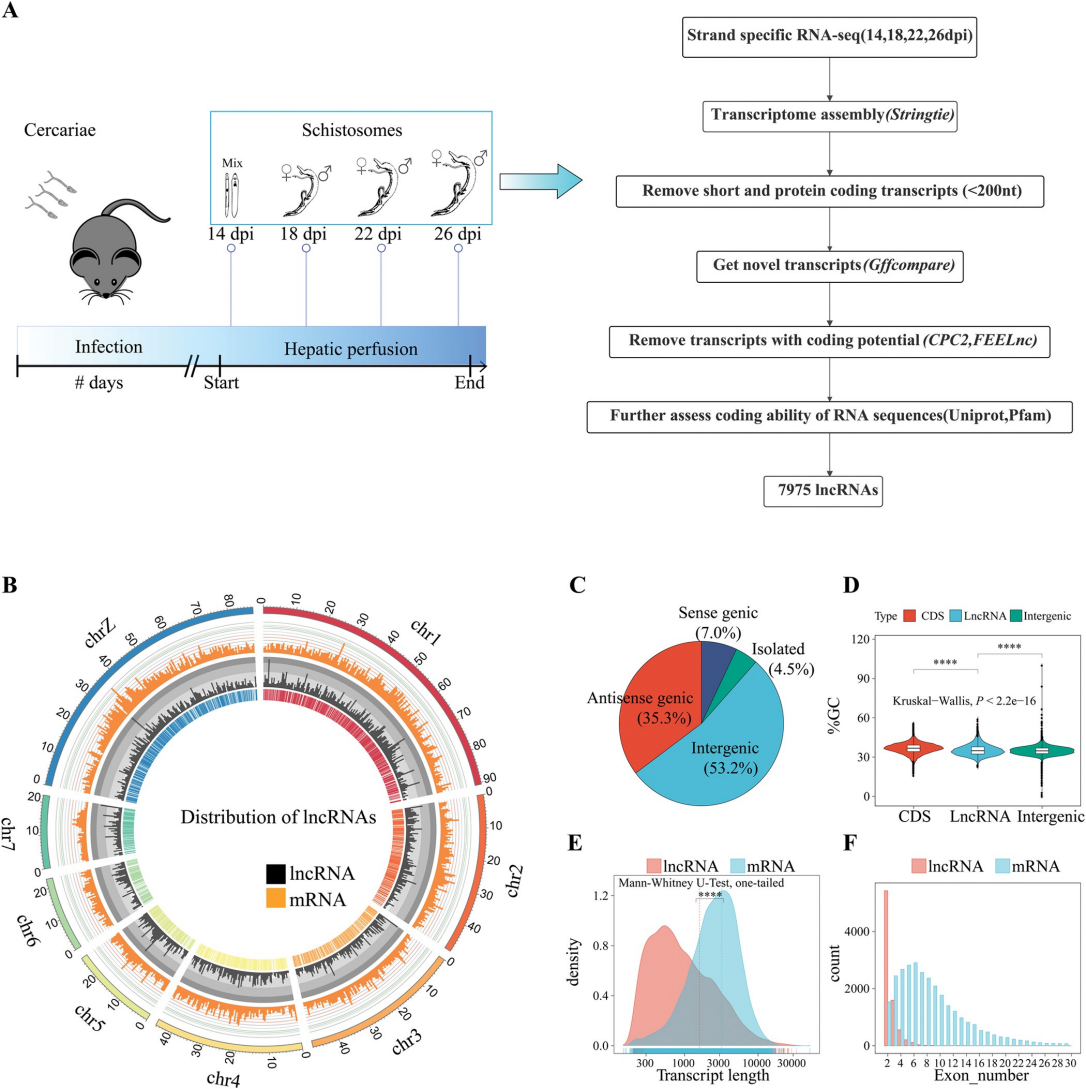
Identification and Characterization of lncRNAs in *S*. *japonicum*. (A) The schematic diagram of schistosomes at four-time points and the bioinformatics workflow for lncRNA prediction. See the description in Methods for details. The cartoon mouse was modified from a public domain image by Creazilla (https://creazilla.com/nodes/19261-cartoon-grey-mouse-clipart). (B) Circos plot showing the chromosomal distribution of lncRNAs. The histogram indicates the abundance of lncRNAs(black) and mRNAs(orange) in physical bins of 10 Kb for each chromosome. Lines located in the innermost circle mark the genomic localization of lncRNAs. (C) The classification of lncRNAs into four categories (intergenic, antisense genic, sense genic, and isolated) according to the relationships between lncRNAs and their adjacent mRNA. (D) GC content of lncRNA transcripts (lncRNA), protein-coding sequences (CDS), and intergenic sequences (Intergenic). The respective average values for the GC content of LncRNA, CDS, and Intergenic regions are 35.39%, 36.59%, and 34.83%. Statistical analysis was performed using the Kruskal-Wallis test with Dunn’s multiple comparisons, ****, *P*-value < 0.0001. (E) Lengths distribution of lncRNA and mRNA transcripts. (F) Numbers of exons in lncRNA and mRNA genes.

To identify lncRNAs expressed in *S*. *japonicum* throughout the male-female pairing and sexual development, we harvested worms at four-time points (14, 18, 22, 26 dpi) and constructed 21 libraries of poly(A)+ RNA for strand-specific RNA sequencing (ssRNA-seq) on the Illumina Nova 6000 platform (Fig 1A). After filtering the low-quality and contaminated reads as well as adapters, we obtained a total of 634.3 million clean reads that were mapped to the newest reference genome of *S*. *japonicum* (*Sj*V3) [34] with alignment rates ranging from 74.8% to 89.0% (S1 Table).

These mapped reads were then assembled to reconstruct the *S*. *japonicum* transcriptome using StringTie [35], resulting in 74,699 unique transcripts greater than 200 nt in length. To acquire the lncRNAs from these transcripts, we not only removed the transcripts that overlapped with protein-coding genes in the same strand or mono-exonic transcripts but also filtered all novel transcripts with coding potential. Only transcripts that successfully passed all filters were considered putative lncRNAs. Overall, we identified 7,975 lncRNAs from 4,917 genomic loci (S1 Fig and S2 Table).

We further characterized the features of these lncRNAs. 94.8% of lncRNAs were mapped to eight chromosomes of *S*. *japonicum* (Fig 1B) and chromosome 1 contained the largest number of lncRNA loci, which was 1,201. In addition, 973 lncRNAs were located on the Z chromosome (S2 Table). By using the sliding-window-based classifier module of the tool FEELnc with a maximum window extension of 100,000 base pairs (bp), the identified lncRNAs could be classified into four major categories, of which 4,239 (53.2%) lncRNAs were located in the intergenic region, i.e. lincRNAs (long intergenic noncoding RNAs), 2,815 (35.3%) lncRNAs were located in the antisense genic region, i.e. NAT-lncRNAs (long non-coding natural antisense transcripts), 560 (7%) lncRNAs on the sense strand of the protein-coding genes and 361 (4.5%) lncRNAs located in the intergenic region with more than 100 kb away from the protein-coding genes (Fig 1C). For the GC content, lncRNA sequences exhibited a lower value than the protein-coding sequences (CDS) but a higher value than the intergenic region sequences (Fig 1D). The respective average values for the GC content of lncRNA, CDS, and Intergenic regions are 35.39%, 36.59%, and 34.83%, respectively. The average length of lncRNAs was 1,592 nt, which was significantly shorter than that of coding RNA (Fig 1E, *P*-value < 0.0001, Mann-Whitney U-Test, one-tailed). Furthermore, the number of exons of these identified lncRNAs was less than that of coding RNA (Fig 1F).

Notably, compared with the previously annotated *S*. *japonicum* lncRNAs by Maciel *et al*. [27] using published non-strand-specific sequencing data, about 73.4% of the lncRNAs we identified were consistent with those in the previous report (BLASTN e-value < 1.0e^-5^), revealing 2,122 novel lncRNAs in this work. Among them, nearly half (1,038) were NAT-lncRNAs, which was much higher (48.9%) than the proportion of NAT-lncRNAs in the whole lncRNAs (35.3%) (S2 Table and S1 File). Together, these results not only provide a general overview of the lncRNAs in *S*. *japonicum* but also improve the annotation of lncRNAs, especially expanding the annotation of natural antisense lncRNAs in the genome.

### Dynamic expression of lncRNAs during the sexual development of *S*. *japonicum*

**Fig 2:**
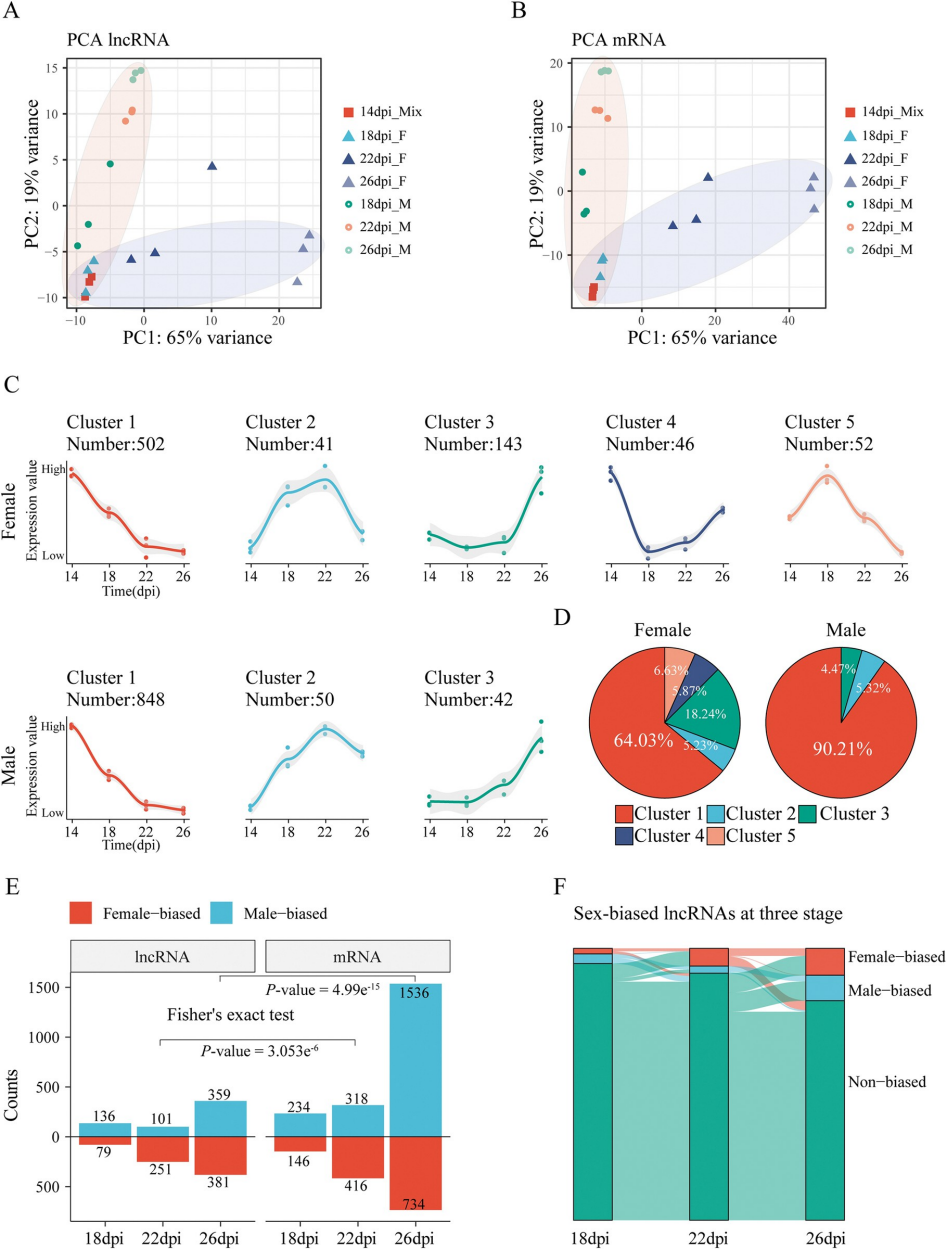
Dynamic expression profiles of lncRNAs during schistosome reproductive development. (A-B) Principal component analysis (PCA) of 21 distinct samples across four-time points based on normalized lncRNAs (A) and mRNAs (B) expression levels. Male and female samples were mainly located in regions indicated by blue and red shading. (C) Clustering of major expression patterns of dynamic lncRNAs during reproductive development in females (up) and males (down). Lines estimated through LOESS regression; 95% confidence interval shown in grey. (D) The proportion of lncRNAs in each cluster to the total dynamic lncRNAs in male and female worms. (E) The bar plot represents the count of sex-biased lncRNAs and mRNAs at 18, 22, and 26 dpi. The categories were divided into female-biased (Log_2_FC ≥ 1, adjusted *P*-value < 0.05) and male-biased (Log_2_FC ≤ -1, adjusted *P*-value < 0.05) genes. A significant difference was observed between the counts of sex-biased lncRNAs and mRNAs (*P*-value < 0.001, Fisher’s exact test). (F) Sankey diagram showing the transition of female-biased lncRNAs (red), male-biased lncRNAs (blue), and non-biased lncRNAs (green) between 18, 22, and 26 dpi. The proportion of each class is represented by the size of the rectangle.

To provide an overview the lncRNA profiles in male and female schistosomes during their interplay and sexual development, we performed principal component analysis (PCA) on our ssRNA-seq dataset (Fig 2A and 2B). The analysis revealed clear separation of samples based on both lncRNA expression patterns (Fig 2A) and mRNA expression patterns (Fig 2B), indicating that the lncRNA profiles in males and females become increasingly dissimilar as they progress through maturation from 14 to 26 dpi. Additionally, the Pearson correlation analysis exhibited these 21 ssRNA-seq samples were well clustered by gender and stage (S2 Fig).

We then evaluated the dynamics of lncRNAs in both male and female *S*. *japonicum* during their reproductive development. We observed a gradual decrease in the average number of expressed lncRNAs (TPM value greater than 1 in at least two biological replicates) in both sexes from 14 to 26 dpi (S3A Fig). Using the maSigPro regression-based method, we identified 784 lncRNAs in females and 940 lncRNAs in males that exhibited significant changes in expression over time during sexual maturation. Notably, nearly half of the protein-coding genes were also found to be dynamic in each gender (S3B Fig). Generally, these dynamic lncRNAs were expressed at higher levels than those non-dynamic lncRNAs (S3C Fig).

To gain an overview the expression patterns of these dynamic lncRNAs during these developmental stages, we performed clustering analysis, resulting in the identification of five clusters in females and three clusters in males (Figs 2C and S3D). Strikingly, a majority of the differentially expressed lncRNAs exhibited a consistent pattern of decreasing expression over time in both sexes (Fig 2C and 2D). Specifically, 64.03% of the lncRNAs in females and 90.21% of the lncRNAs in males showed a decreased in expression from 14 to 26 dpi (Fig 2D).

Next, we investigated the sex-biased lncRNAs in *S*. *japonicum*. We identified sex-biased lncRNAs (|Log_2_FC| ≥1, adjusted *P*-value < 0.05) by pair-wise comparison of male and female samples at the same stage using DESeq2(S3 Table). As shown in S4A Fig, the volcano plots displayed the differentially sex-biased lncRNAs identified at each stage. The number of sex-biased lncRNAs increased during sexual development, peaking at 26 dpi with 359 male-biased and 381 female-biased lncRNAs (Fig 2E). This pattern is closely related to the biology of *S*. *japonicum*, where the difference between male and female becomes more pronounced at 26 dpi when they reach sexual maturity. Furthermore, we observed a similar increase in the number of mRNAs with sex-based bias, paralleling the pattern observed for sex-biased lncRNAs (Fig 2E). Tracking the transitions between sex-biased and non-biased lncRNAs at these three time points, we discovered the occurrence of switching between these two types of lncRNAs (Fig 2F). In addition, we found that only a limited number of lncRNAs displayed a consistent sex-biased expression pattern, which we defined as sex-specific lncRNAs. A hierarchical clustering heatmap was generated to illustrate the expression patterns of these sex-specific lncRNAs at each sample, categorized by sex and stage, revealing 23 female-specific and 44 male-specific lncRNAs, respectively (S4B Fig). Notably, 8 female-specific lncRNAs and 20 male-specific lncRNAs were located on Z-chromosomes (S2 Table).

Taken together, these findings highlight that, similar to mRNAs, the expression of lncRNAs undergoes sex-specific changes at distinct time points ranging from 14 to 26 dpi. This observation strongly suggests that lncRNAs likely exert a significant influence on the reproductive development and subsequent egg production of schistosomes.

### Exploring the function of lncRNAs in the sexual development of *S*. *japonicum* through co-expression network analysis

**Fig 3:**
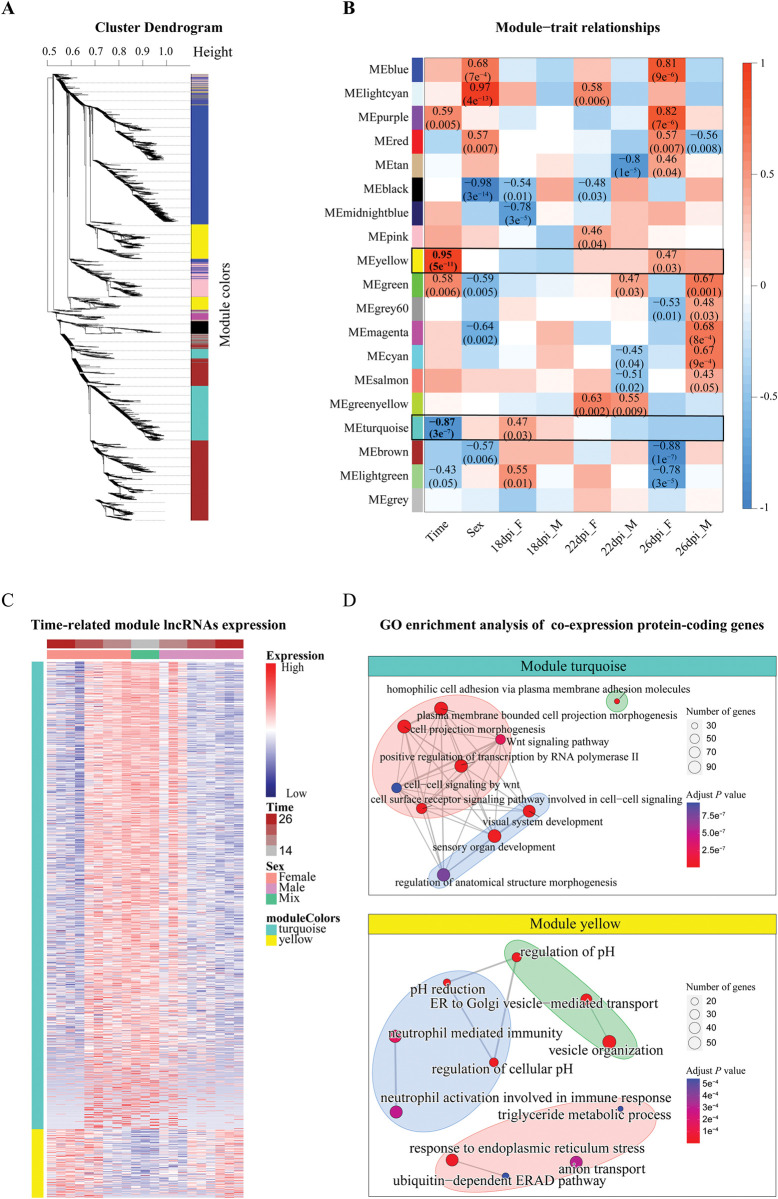
Weighted gene correlation network analysis (WGCNA) identifies lncRNAs-mRNA co-expression network modules. (A) The dendrogram shows 19 lncRNAs-mRNA co-expression modules identified using WGCNA. (B) The heatmap illustrates the correlation between the identified modules and various biological traits. Each column represents a trait, while each row corresponds to a module eigengene. The strength and direction of the association between each trait and eigengene are indicated by a Pearson’s correlation coefficient, with the corresponding *P*-value provided in parentheses. The color gradient within each cell reflects the correlation, with blue signifying a strong negative correlation and red indicating a strong positive correlation. (C) Heatmap of lncRNAs in the turquoise and yellow modules during 14–26 dpi in male and female worms. Red and blue represent high and low gene expression levels, respectively. (D) Gene ontology (GO)) enrichment of the coding genes in turquoise and yellow modules. Color intensity represents the magnitude of -log_10_(adjusted *P*-value) (Fisher’s exact test). Only GO terms with adjusted *P*-value < 10^−4^ are shown.

To investigate the potential roles of lncRNAs in the reproduction and development of *S*. *japonicum*, we utilized the weighted gene coexpression network analysis (WGCNA) to construct a lncRNAs-mRNAs co-expression network [36]. By selecting the optimal soft threshold (*β* = 12), we obtained 19 main co-expression modules that each represented a unique expression pattern (Figs 3A, S5 and S4 Tables). Among these modules, the turquoise module emerged as the largest, encompassing a total of 2,304 genes, while the light-green module stood as the smallest, comprising only 48 genes. Notably, lncRNA genes contributed to varying extents, ranging from 8% to 85%, in the composition of each module (S5 Fig), underscoring the heterogeneous involvement of lncRNAs in co-expression modules. By calculating eigengene values that represent the gene expression profiles of each module, we further identified time- and sex-related modules (S5 Fig). To assess the relationship between each module and time as well as sex, we conducted a Pearson correlation analysis (Fig 3B). Consequently, two modules, turquoise and yellow, exhibited significant correlations with time, indicating their pronounced involvement in the developmental process across genders. The turquoise module, which comprised 1,139 lncRNAs and 1,165 mRNAs, displayed a significant negative correlation with time (*R* = -0.87, *P*-value = 3e^-7^). In contrast, the yellow module, consisting of 169 lncRNAs and 1,117 mRNAs, showed a substantial positive correlation with time (*R* = 0.95, *P*-value = 5e^-11^). A heatmap visualizing the expression patterns of lncRNAs in these two modules over time clearly demonstrated their opposite trends (Fig 3C). Specifically, in the largest turquoise module, the expression of lncRNAs exhibited a decreasing trend from 14 to 26 dpi, with the most substantial changes occurring during the transition from 18 to 22 dpi. These findings suggest that a significant majority of lncRNAs are down-regulated during the sexual development of *S*. *japonicum*.

The GO analysis on the mRNAs of the lncRNAs-mRNAs co-expression network in the turquoise module demonstrated enrichment in several biological processes (BP). These processed include homophilic cell adhesion of plasma membrane adhesion molecules, positive regulation of transcription RNA polymerase II, plasma membrane bounded cell projection morphogenesis, cell surface receptor signaling pathway involved in cell-cell signaling, Wnt signaling pathway. Additionally, GO terms related to developmental regulation, such as sensory organ development, visual system development, regulation of neurogenesis, epidermis development, regulation of muscle tissue development, and sex differentiation, were also enriched (Fig 3D and S5 Table). In contrast, the mRNAs in the co-expression network of the yellow module that up-regulated during the reproductive development of both sexes were highly enriched in regulation of pH, vesicle-mediated transport, and anion transport, which may be associated with the enhanced exchange of various substances both in males and females during their interplay.

Notably, 10 out of the 17 analyzed modules showed significant enrichment in at least one GO term. For modules without significant GO term enrichment, we inferred potential functions based on co-expression hub genes. For example, *sperm-egg fusion protein Juno (IZUMO1R*, *Sjc_0005723)* was identified as a hub-gene within the lightcyan module due to its strong correlation with sex-related traits. This gene encodes the IZUMO1 receptor, which is present on the cell surface of the oocyte and plays a crucial role in species-specific gamete recognition and fertilization [37–39]. These findings provide insights into the potential functional roles of lncRNAs in different co-expression modules.

Overall, we successfully constructed a comprehensive lncRNA-mRNA co-expression network throughout the sexual development of *S*. *japonicum*. By clustering lncRNAs into modules that represent distinct expression patterns, we not only predicted co-expression networks in which lncRNAs are potentially involved in regulation but also annotated the biological processes associated with these gene regulatory networks at different time points. Moreover, we identified key lncRNAs that occupy important positions within the regulatory network. These findings contribute to a better understanding the functions of lncRNAs in *S*. *japonicum* and shed light on the transcriptional regulatory mechanisms governing sexual development.

### Functional characterization of hub-lncRNAs in time-related yellow module

**Fig 4:**
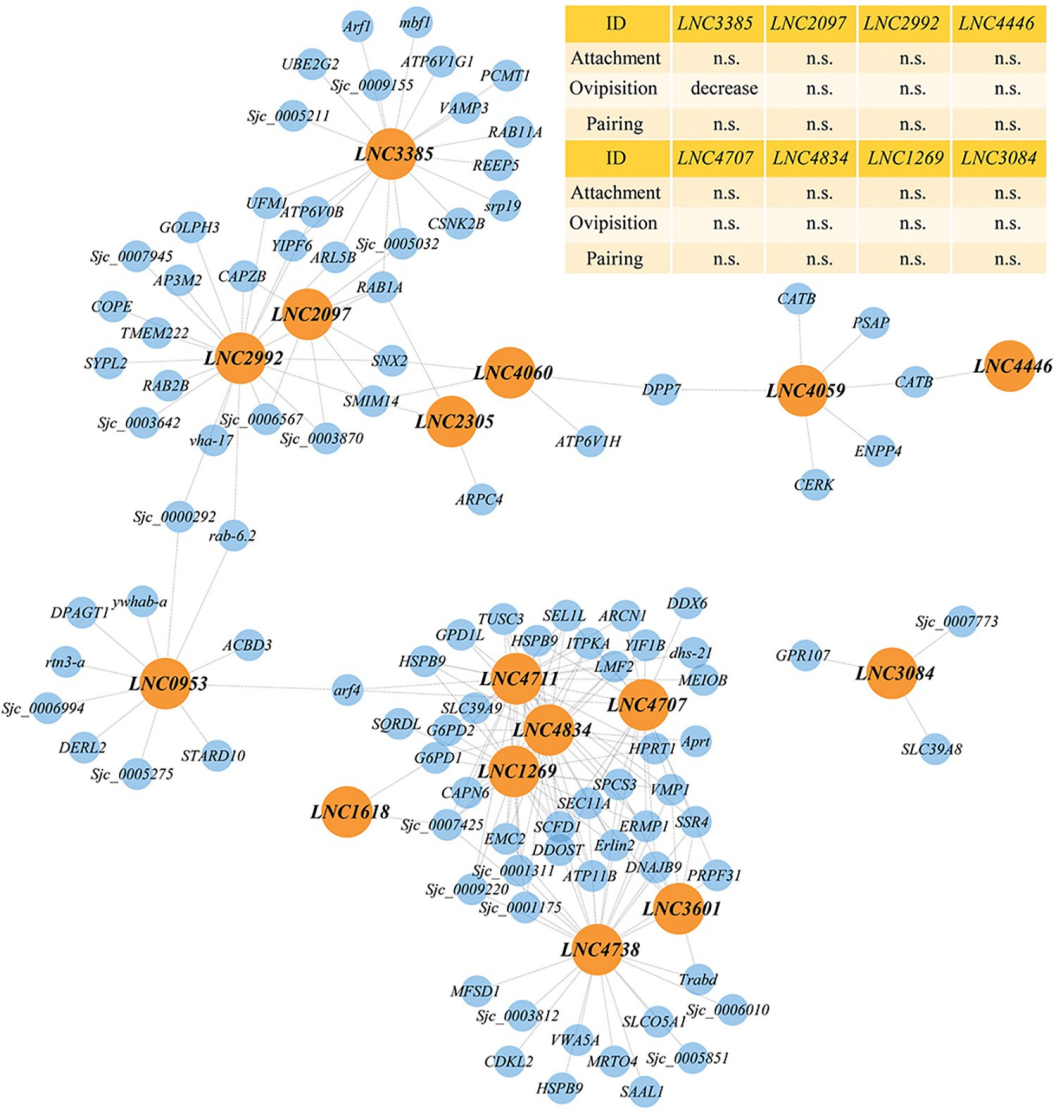
Subnetwork of hub lncRNAs extracted from yellow module. The figure shows the network of interconnections between hub lncRNAs and their co-expression mRNAs (TOM > 0.2) within the yellow module. Hub-lncRNAs are represented by orange circles, while mRNAs are depicted as blue circles. The table on the upper right corner shows the performance of *S*.*japoniucm* after RNAi of selected lncRNAs.

To identify potential regulatory lncRNAs involved in the sexual development of *S*. *japonicum*, we focused on the yellow module where lncRNAs exhibited upregulation during male-female pairing (Fig 2C). Initially, we identified hub-lncRNAs within this yellow module using the signedKME function in the R package WGCNA. Subsequently, we selected all hub-lncRNAs with |KME| > 0.75 and TOM > 0.2, along with their co-expressed mRNAs, to establish a subnetwork of hub-lncRNAs. This subnetwork formed a core regulatory subnetwork comprising 16 lncRNAs and 100 co-expressed mRNAs (Fig 4 and S6 Table). Among these protein-coding genes, some were determined to play a role in in germ cell development, such as *eledh* (*eled*), *meiosis specific with OB domains (MEIOB)* and *START domain-containing protein 10 (Stard10)* [40,41]. There were also genes involved in protein transport, including *derlin-2(DERL2)*, *golgi resident protein GCP60 (ACBD3)*, *ras-related protein Rab-6A (RAB6A)*, *vacuole membrane protein 1(VMP1)*, *vesicle-associated membrane protein 3 (VAMP3)*, *reticulon-3-A(RTN3)* and *protein sel-1 homolog 1 (SEL1L)* [42–47].

**Fig 5:**
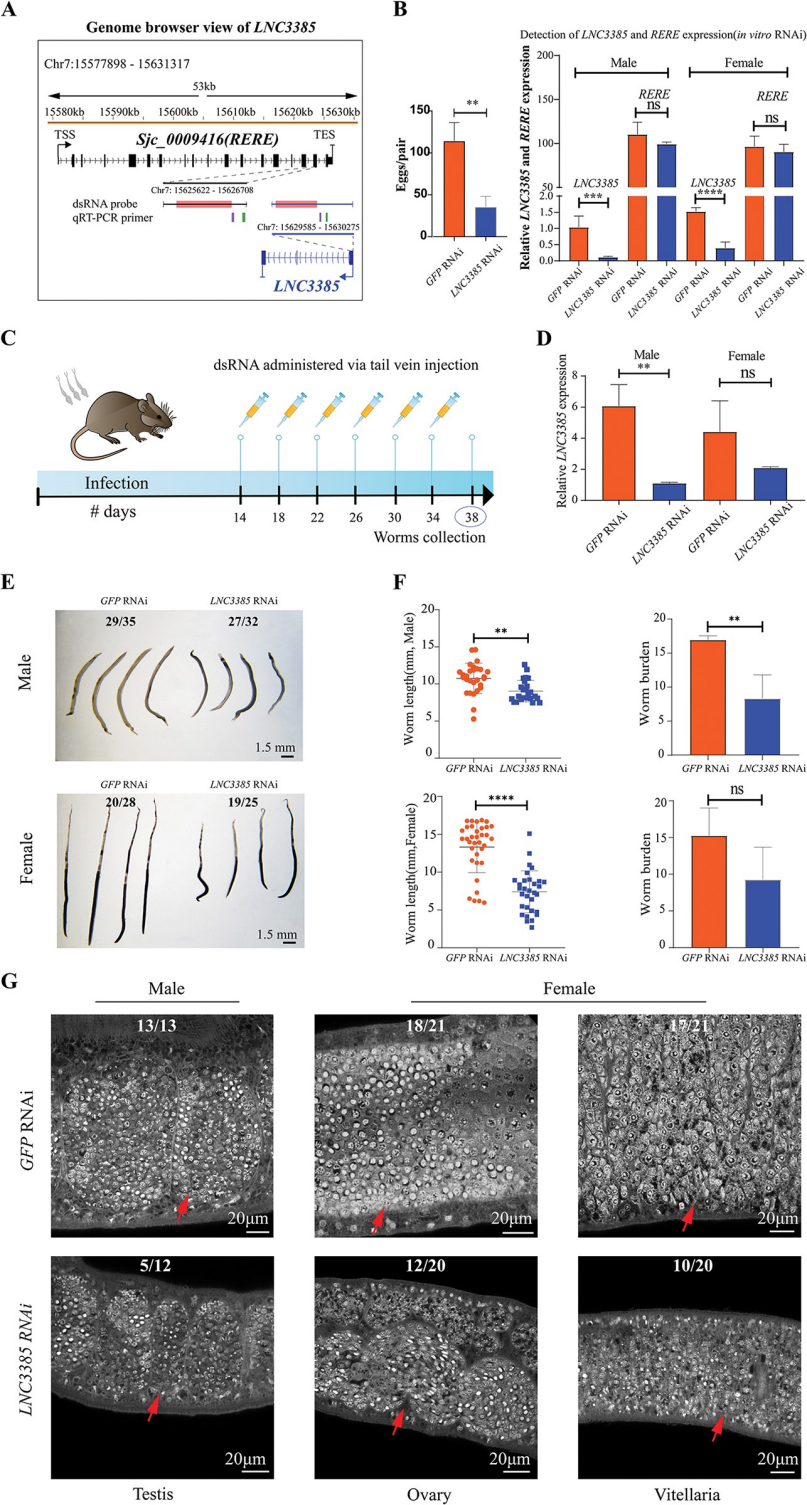
A natural antisense lncRNA *LNC3385* regulates the development of *S*. *japonicum*. (A) Genome browser view of *LNC3385* and *RERE* (*Sjc_0009416*). The gene structure model is shown below the chromosomal coordinate axis. The central red block denotes the region targeted by the *LNC3385* and *RERE* dsRNA probes. The qRT-PCR primer lines indicate the genomic positions where primers for these genes are designed: purple represents the forward primer, and green represents the reverse primer. (B) Silencing *LNC3385* led to decreased egg production of female worms *in vitro*. Left, the number of eggs per pair after the treatment of *GFP* or *LNC3385* dsRNA for 8 Days; Right, qPCR analysis showing the relative expression level (mean ± SE, n = 3) of *LNC3385* and *RERE* in males and females after RNAi which shows a 74.1% decrease in *LNC3385* expression levels in females and an 89.2% decrease in males, as compared to *GFP*-treated controls. (C) Schematic diagram illustrating the *in vivo* RNAi experiments conducted post cercariae infection. Mouse image modified from Wikimedia Commons (https://commons.wikimedia.org/wiki/File:Vector_diagram_of_laboratory_mouse_(black_and_white).svg, CC BY-SA 4.0). Syringe icon modified from Wikimedia Commons (https://commons.wikimedia.org/wiki/File:Noto_Emoji_Oreo_1f489.svg, Apache License 2.0). (D) Relative expression level (mean ± SE) of *LNC3385* in RNAi-treated parasites by qPCR analysis. *In vivo* RNAi resulted in an 81.9% decrease in *LNC3385* expression in male worms, while females showed a similar reduction that was not statistically significant. Three biological replicates were performed. (E) Brightfield microscopy of male and female parasites from *GFP* and *LNC3385* dsRNA treated groups. (F) Worm length (left) and worm burden (right) of the parasites recovered at 38 dpi in *GFP* and *LNC3385* RNAi groups. (G) Morphological changes of male and female worms in the reproductive system after *in vivo* RNAi of *LNC3385*. * *P*-value < 0.05; ** *P*-value < 0.01; *** *P*-value < 0.005; **** *P*-value < 0.001; n.s. not statistically significant.

To further investigate the potential functional roles of these hub-lncRNAs in *S*. *japonicum*, we selected eight candidate lncRNAs based on their average expression levels and network connectivity within the WGCNA co-expression network. Subsequently, we conducted RNAi experiments *in vitro* to examine their effects (Fig 4). Assessing the physical activities, such as locomotion and pairing, of the dsRNA-treated parasites, we observed no discernible phenotypic changes associated with any of the eight lncRNAs (Fig 4 upper right corner). We then envaulted their effect on egg production and found that silencing *LNC3385*, a NAT-lncRNA that was transcribed from the antisense strand of the *Arginine-glutamic acid dipeptide repeats* (*RERE*, *Sjc_0009416*) gene (Fig 5A), lead to a dramatic decrease in egg-laying compared to the control group (Fig 5B). Although the expression levels of the remaining seven lncRNAs were effectively reduced, they did not demonstrate a considerable influence on schistosome egg production (S6 Fig). It is noteworthy that the expression pattern of *RERE*, the cognate gene of *LNC3385*, exhibited an opposite trend from 14 to 28 dpi (S7A–S7C Fig). We further examined the expression of *LNC3385* from our ssRNA-seq dataset by *in situ* hybridization which revealed the localization of *LNC3385* throughout the entire body of the worms, with its expression gradually upregulated during the developmental process (S7D Fig). Additionally, to investigate the relationship between *LNC3385* and *RERE*, we examined the expression levels of *RERE* in both sexes after performing *in vitro* RNAi knockdown of *LNC3385* using RT-PCR. However, our results indicated only a slight non-significant decrease in the expression level of *RERE* (Fig 5B). Similarly, when we conducted *RERE* knockdown experiments in both female and male worms, we observed no significant change in the expression level of *LNC3385* (S7E Fig). These findings suggest that the reduction in *RERE* expression is not necessary for the observed decrease in egg-laying phenotype, indicating that *LNC3385* may exert its effects in a *trans*-regulatory manner.

Taken together, these data highlight the potential role of the NAT-lncRNA *LNC3385* in regulating egg laying in schistosomes.

### *LNC3385* regulates reproductive development in *S*. *japonicum*

Since the reproductive organs of adult *S*. *japonicum* could degenerate *in vitro* [48], we further performed *in vivo* RNAi on *LNC3385* in mice model by silencing its expression from 14 dpi and collected the worms at 38 dpi (Fig 5C). As indicated in Fig 5, knocking down *LNC3385* not only led to a significant decrease in body length of both female and male worms compared to the *GFP*-treated group, but also strongly reduced the worm burden of male parasites (Fig 5D–5F). To explore whether *LNC3385* RNAi affects the normal development of the schistosome reproductive system, we examined the morphological changes of male and female sexual organs using confocal laser scanning microscopy (CLSM). The *LNC3385* RNAi-treated male worms failed to develop mature testis, which contained smaller testicular lobes, rare mature spermatogonia and spermatocytes. Similarly, most of the *LNC3385* RNAi female worms had undeveloped ovaries and vitellaria (Figs 5G and S8). Additionally, the HE staining and Masson’s staining displayed reduced liver pathology with fewer eggs and granulomas in the mice treated with *LNC3385* dsRNA (S9A–S9D Fig). The size of the surrounding granuloma area related to individual eggs significantly decreased in livers from the *LNC3385* RNAi group, indicating a decrease in the viability of the eggs (S9E Fig). These data suggested that *LNC3385* might act as an important regulator in the survival, development and reproduction of *S*. *japonicum* in its definitive host.

### Transcriptome analysis reveals a potential regulatory network controlled by *LNC3385*

**Fig 6:**
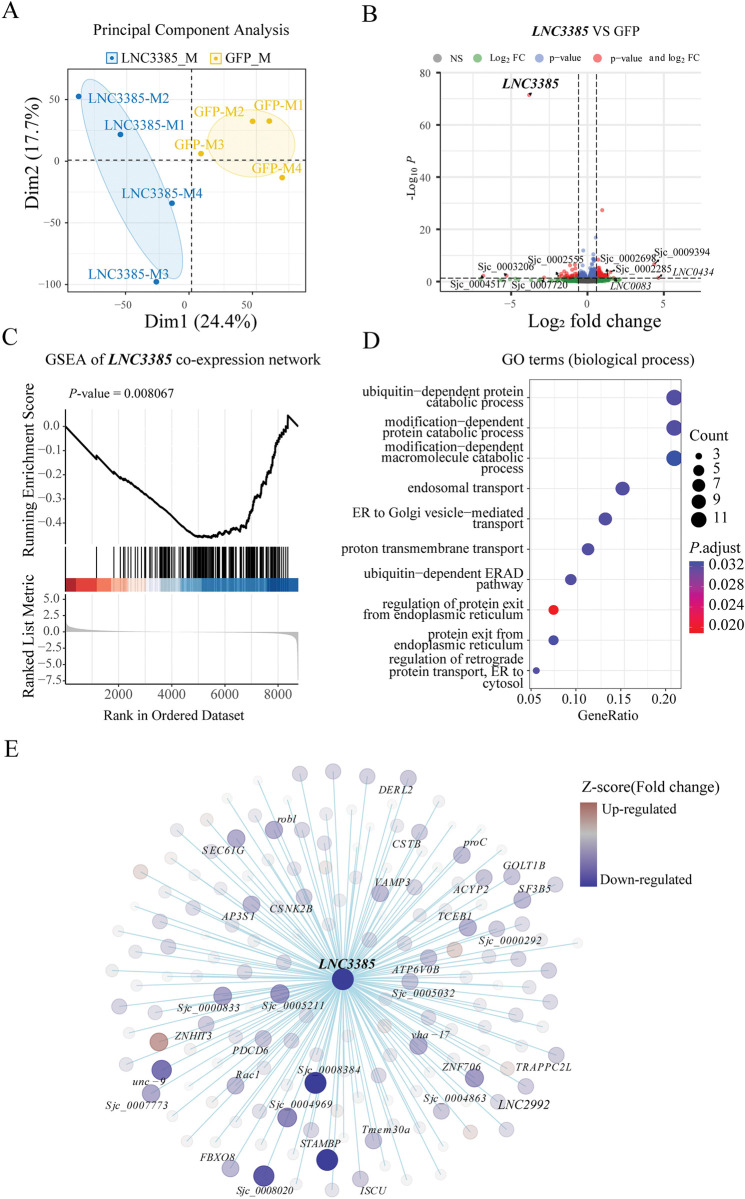
Transcriptomic and gene network analysis following *LNC3385* RNAi *in vitro*. (A) Principal component analysis (PCA) of the transcriptome of *LNC3385* or *GFP* dsRNA treated worms. (B) Volcano plot of differentially expressed genes (DEGs). DEGs were defined as |log_2_ (fold change)| ≥ 1 and adjusted *P*-value< 0.05. (C) Gene set enrichment analysis (GSEA) showed the downregulation of genes in the *LNC3385* co-expression network (TOM > 0.1) in the *LNC3385* RNAi group. (D) Gene Ontology analysis of core enrichment genes, which contribute most to the enrichment result, within the *LNC3385* co-expression gene set. (E) The diagram represents a hypothesized gene network regulated by *LNC3385* (TOM > 0.1). Each node represents a gene and the color indicates the expression level. Genes with a fold change < -0.15 and *P*-value < 0.05 are labeled.

To further determine the role of *LNC3385* in *S*. *japoncium*, we performed RNA-seq on male parasites after *GFP* and *LNC3385* RNAi treatment *in vitro*. Principal component analysis (PCA) showed the separated profiles of the *GFP* and *LNC3385* RNAi samples (Fig 6A). Differential gene expression analysis revealed that knockdown *LNC3385* resulted in the upregulation of 364 genes and the downregulation of 157 genes (|Log_2_FC| > 0, adjusted *P*-value < 0.05). Notably, 18 genes displayed over 2-fold upregulation, while 22 genes exhibited over 2-fold downregulation (Figs 6B, S10 and S7 Table).

To identify potential co-expressed genes regulated by *LNC3385*, we selected all genes with a TOM value greater than 0.1 from the yellow module. This set of genes was used for Gene Set Enrichment Analysis (GSEA), which revealed a significant downregulation of this gene set following *LNC3385* RNAi treatment (Fig 6C). We found that the core enriched genes were functionally associated with biological processes such as ubiquitin-dependent ERAD pathway, ER to Golgi vesicle-mediated transport, and proton transmembrane transport. Notably, these Gene Ontology (GO) terms were also enriched in the entire yellow module as identified previously (Figs 6D and 3D). Through this analysis, we successfully identified a regulatory network wherein *LNC3385* plays a pivotal role, leading to substantial downregulation of numerous putative co-expressed genes. Noteworthy genes in this network included *STAMBP*, *unc-9* and *vha-17*(Fig 6E). These genes, which were likely regulated by *LNC3385*, functioned closely associated with de-ubiquitination, ion transmembrane transport and vesicular transport. Collectively, these results demonstrate that the knockdown of *LNC3385* changed the expression of genes predominantly within the module controlled by *LNC3385*, consequently affecting the development and reproduction of *S*. *japonicum*.

## Discussion

The reproductive development is one the most fascinating biology of schistosomes since the females rely on pairing with males to launch their sexual development. The role of lncRNA in this process is not yet clear. In this study, we employed strand-specific RNA-seq to identify 7,975 lncRNAs in *S*. *japonicum* during the sexual development of both sexes and provided their dynamic expression profiles. We then constructed a weighted lncRNA-mRNA co-expression network. By performing RNAi screening on several hub-lncRNAs that were upregulated from 14 to 26 dpi, we identified *LNC3385* as a functional lncRNA that was essential for the survival, development, and egg production of *S*. *japonicum*.

Comprehensive annotation of lncRNAs is fundamental for downstream functional studies [49]. In previous studies, Liao *et al*. [50] and Maciel *et al*. [27] identified the lncRNAs of *S*. *japonicum* using published non-strand specific RNA-seq datasets. Here, we identified and annotated lncRNAs on a chromosome level refereeing to our recently published third-generation genome of *S*. *japonicum* (*Sj*V3) [34] using strand-specific RNA-seq, a technique that could retain strand information of reads and provide a more accurate estimate of antisense transcript expression [51]. Notably, compared to the previously identified lncRNAs by Maciel *et al*. [28], we revealed 2,122 novel lncRNAs and almost half of them (48.9%) were NAT-lncRNAs (S1 File and S2 Table).

Both male and female schistosomes undergo dramatic changes in the coding genes during their pairing and sexual development [7]. Our data demonstrated that lncRNAs also change dramatically throughout this process. In detail, the expression levels of most lncRNAs are gradually down-regulated along with the developmental process, especially in male schistosomes (Fig 2C). In addition, similar to mRNA, the number of sex-biased lncRNAs increased along with their maturation (Fig 2E and 2F). As described by Silveira *et al*., several female-biased lncRNAs were associated with the maintenance of female vitellaria [33]. These data suggest that lncRNAs may also participate in the sexual development of schistosomes.

Unlike protein-coding genes, lncRNAs are generally not conserved in their sequence and secondary structures [52,53], making it difficult to predict their functions. WGCNA is one of the most widely used methods for identifying highly related gene modules that may be involved in similar biological functions [36,54]. Using this approach, we constructed 19 lncRNAs-mRNA co-expression modules associated with various developmental time points and sex, and revealed lncRNAs-mRNA gene regulatory networks that may play potential regulatory roles individually or cooperatively at different developmental stages (Figs 3 and S5). For instance, the turquoise and yellow modules were highly correlated with development time, while the black and lightcyan modules were highly correlated with sex (Fig 3). In addition, we provided a more comprehensive functional prediction of lncRNAs based on the GO terms enrichment analysis of the protein-coding genes in each module (S5 Table). These results further support the hypothesis that lncRNAs play an important regulatory role in the reproductive development of *S*. *japonicum*. It is important to highlight that our research primarily focused on investigating the lncRNAs within the yellow modules. However, it is equally important to explore other lncRNAs in this module as well other networks for a comprehensive understanding of their role and significance.

Although there are over 10,000 lncRNAs in schistosomes, a few of them have been functionally characterized [29,33,55]. However, no regulatory mechanism of these lncRNAs has been uncovered to explain their linked worm phenotype. Here we identified a lncRNA by RNAi that silencing *LNC3385* led to reduced egg production of adult females *in vitro* (Figs 4 and 5B). Due to the limitations of the *in vitro* conditions, we further knocked down *LNC3385 in vivo* and observed more severe phenotype, including decreased egg laying, reduced size of both male and female worms in length, hindered reproductive organs, and lower worm burden in male worms (Figs 5C–5E, S8 and S9). The difference in the phenotypes between *in vitro* and *in vivo* RNAi is expectable since several key elements are missing in the culture conditions, such as the optimal nutritional supports for worm growth and development and host immune responses. Nevertheless, these findings reveal *LNC3385* is functional in schistosomes, which may play a role in schistosome development and reproduction. Subsequent comparative transcriptomic analyses of worms subjected to *in vitro* dsRNA treatments with *GFP* and *LNC3385* revealed that *LNC3385* downregulation notably suppressed numerous putatively co-expressed genes, including *STAMBP*, *unc-9* and *vha-17* (Fig 6E), thereby hindering normal growth and development of *S*. *japonicum*.

The functions of lncRNAs can be broadly classified into those that regulate local chromatin structure and/or gene expression *in cis* versus those that perform cellular functions away from the site of transcription *in trans* [15]. Despite observing a significant negative correlation in their expression during development (S7A, S7B and S7C Fig), our data showed that knocking down either *LNC3385* or *RERE* did not have any significant impact on the expression of the other gene (Figs 5B and S7E). We speculated that *LNC3385* may not influence the expression of its nearby gene *RERE in cis*. The transcriptome analysis of the male worms after knocking down *LNC3385* indicated that *LNC3385* plays a key role in regulating its co-expressed genes (Fig 6E). Indeed, at present, we do not know how *LNC3385* could regulate the expression of such genes.

Considering lncRNAs may modulate gene transcription via interactions with chromatin, we conducted a comprehensive investigation into the impact of *LNC3385* RNAi on chromatin accessibility in schistosomes using ATAC-seq. Notably, our results demonstrated the expected profile of ATAC-seq in terms of fragment size and transcription start site (TSS) enrichment (S11 Fig and S8 Table). Intriguingly, we observed a prominent chromatin accessibility signal on one of the exons of *LNC3385* (S12A Fig), indicative of its potential role as an enhancer-associated lncRNA (elncRNA) [56]. Additionally, a genome-wide analysis examining the overlap between lncRNAs and ATAC peaks generated a curated list of candidate elncRNAs (S12B Fig). Although we didn’t detect significant global changes in chromatin accessibility between *GFP* RNAi and *LNC3385* RNAi worms, chromatin accessibility at a few ATAC peaks did exhibit significant changes with 82 down-regulated and 56 up-regulated out of total 18,971 peaks (S13A Fig and S9 Table). We noted a significant decrease in accessibility at the promoter region of a UPF0506 domain-containing protein named *Sjc_0008020* following *LNC3385* RNAi treatment. This observation came from an examination of changes in the ATAC-seq signal surrounding *LNC3385’s* predicted target gene as identified from RNA-seq data (S13B and S13C Fig and S10 Table). However, the precise mechanism by which *LNC3385* affects the chromatin accessibility of *Sjc_0008020* remains unknown. Nevertheless, these compelling findings strongly suggest that *LNC3385* may have the capacity to translocate to proximal gene loci after transcription, subsequently modulating gene expression via epigenetic regulation through chromatin interactions. It also is important to note that, alongside the evident effect of *LNC3385* on chromatin accessibility, further investigations are warranted to shed light on alternative mechanisms underlying changes in other genes. These potential mechanisms are yet to be explored, which will provide valuable insights into the broader regulatory landscape of gene expression in this context.

In summary, we provided a comprehensive annotation of lncRNAs and mapped the lncRNA expression profiles during the reproductive development in both sexes of *S*. *japonicum*. We not only characterized lncRNA expression at the temporal and gender levels but also constructed the lncRNA-mRNA co-expression network. Importantly, we identified a functional lncRNA for the first time in *S*. *japonicum*, which is associated with this parasite’s development and egg production. This study deepens our understanding of the regulatory functions of long non-coding RNAs in the reproductive development of *S*. *japonicum*, highlighting the potential of lncRNAs as therapeutic targets.

## Materials and methods

### Ethics statement

The manipulation of animals was conducted in strict compliance with the animal care and use guidelines established by Fudan University. All procedures were approved by the Animal Care and Use Committee of Fudan University (Fudan IACUC 201802158S) to ensure the ethical and responsible treatment of the animals.

### Infection of mice with *S*. *japonicum*

Six-week-old female C57BL/6 mice were purchased from Slack Laboratory Animal Co., Ltd. (Shanghai, China). The cercariae of *S*. *japonicum* were shed from *Oncomelania hupensis* snails, which were provided by the Department of Vector Control of the National Institute of Parasitic Diseases, Chinese Center for Diseases Control and Prevention, Shanghai. For the collection of RNA-seq samples and *in vitro* RNAi experiments, each mouse was infected with approximately 150 cercariae. For the *in vivo* RNAi experiment, each mouse was infected with 60±2 cercariae.

### Sample collection

The worms for RNA-seq were recovered from mice on specific days after infection (14, 18, 22, and 26 days post-infection, dpi) through hepatic-portal perfusion. The parasites were transferred into sterile DMEM supplemented with 5% Fetal Bovine Serum (FBS) and then separated by sex using light microscopy except the worms obtained on 14 dpi due to the difficulty in distinguishing male and female based on morphology. After 2× washes with DEPC water, the parasites were stored at -80°C for sequencing.

### RNA extraction and sequencing

The total RNA extraction, strand-specific RNA library construction, and RNA sequencing were conducted by the Novagene Technology Corporation (Beijing, China). Briefly, RNA was extracted from worms using a modified phenol-chloroform method and column purification. The integrity and quality of total RNA were examined by a Nanodrop ND-2000 spectrophotometer (Thermo Scientific Inc., USA) and an Agilent 2100 Bioanalyzer (Agilent, USA). Pooled RNA was used to construct a strand-specific RNA-Seq (ssRNA-seq) library using Illumina TruSeq RNA sample prep Kit (Illumina, San Diego, CA, USA) with Ribo-Zero Magnetic kit for rRNA depletion according to the TruSeq RNA Sample Preparation Guide. This library was sequenced on the Illumina nova 6000 platforms to acquire 150 bp paired-end (PE) reads.

### Synthesis of dsRNA

To generate double-strand RNA (dsRNA), sequence-specific primers were designed according to *LNC3385* or the control *green fluorescent protein* (*GFP*). The primer sequences are as follows: *LNC3385*(Forward 5’-TAGATTATGGCAGCTACTTG-3’; Reverse 5’-ACCTTGTGGAGCCTAGAA-3’); *GFP* (Forward 5’-GTCAGTGGAGAGGGTGAAG-3’; Reverse 5’-CTAGTTGAACGGATCCATC-3’).

The T7 promoter sequence TAATACGACTCACTATAGGGAGA was added to the 5’ end of each oligo. The DNA template for dsRNA synthesis was amplified with a 2×Hieff PCR Master MIX (with Dye) kit (YEASON, Shanghai, China) and confirmed by Sanger Sequencing. According to the manufacturer’s instructions. The dsRNA was synthesized using MEGAscript T7 Transcription Kit (Thermo Fisher Scientific, Waltham, MA, USA).

### RNA interference *in vitro*

The parasites were recovered from infected mice on 30 dpi through hepatic-portal perfusion and washed with sterile DMEM supplemented with 10% FBS. After being placed on a 12-well cell culture plate with 5 pairs in each well containing 3 mL DMEM supplemented with 10% FBS, they were maintained at 37°C with 5% CO_2_. D0 represents the first day of the experiment. Worms were treated with 15 μg/mL dsRNA on D0, 2, 4, and 6 for one week. The medium was changed every other day. The eggs produced by the worms are retained from D3. On D8, worms were collected for RNA-seq and qPCR.

### RNA interference *in vivo*

Infected mice were randomly allocated into 2 groups with 4 mice each. Every 0.2 mL saline solution (0.9% NaCl solution) containing 10 μg *LNC3385* or *GFP* dsRNA was injected into the tail vein of infected mice on 14, 18, 22, 26, 30, and 34 dpi, respectively. On 38 dpi, adult worms were harvested through hepatic-portal perfusion and then transferred into sterile DMEM supplemented with 5% FBS. Six worm pairs from each mouse were randomly selected to assess the RNAi efficiency using qPCR. The male and female schistosomes were separated and fixed in alcohol-formalin-acetic acid (AFA) for morphometric analyses with Mayer’s carmalum staining as previously described [57]. Worm length was measured by ImageJ. The reproductive organs of the parasites were visualized using a light microscope (Nikon 80i) and Nikon A1 Laser Scanning Confocal Microscope.

### qRT-PCR (Quantitative Real-time PCR)

All qRT-PCR reactions were performed on a LightCycler 96 (Roche, Basel, Switzerland) using 2× SYBR green qRT-PCR master mix (YEASEN, China) according to the manufacturer’s instructions. Each 20 μL qRT-PCR reaction mixture comprised a 2 μL of cDNA(1:4), 10 μL 2× SYBR green master, 0.8 μL (5 μM) of each primer and 6.4 μL ddH_2_O. The qRT-PCR cycle parameters were as follows: 95°C for 3 min, followed by 40 cycles of 95°C for 15 s, 60°C for 30 s; melt curve analysis ranged from 60°C to 95°C to ensure that the specific product was amplified in each reaction. The 2^−ΔCt^ method was used to calculate the relative expression. The endogenous gene *psmd* (*26S proteasome non-ATPase*) served as an internal control. The primer sequences for qPCR are as follows:

*psmd* (Forward 5’-GCAGCACGACTTCTTCAA-3’; Reverse 5’-GAACTCCAGCAGGACCAT-3’). *LNC3385*(Forward 5’-AGTGCCTGTATGCCAGTT-3’; Reverse 5’-GAAGCCAAGATATTCAAGAAGC-3’); *Sjc-0009416* (Forward 5’-CGATAACCACCAAGTTCAAG-3’; Reverse 5’-CTTATGGAAATGACGACGAA-3’);

### *In situ* hybridization

Whole-mount *in situ* hybridization was performed as previously described [58] with the following modification. The template to synthesize the probe for *LNC3385* using the following primers: sense strand 5’-GTAAGAGCGATGAATAAGTAGG-3’; anti-sense strand 5’-GCGTGGTTGTTAATTGGTT-3’. A T7 promoter sequence (TAATACGACTCACTATAGGGAGA) was added to the 5’ end of the anti-sense oligo. A 100 ng/mL probe was used in the hybridization buffer. All labeled parasites were cleared in 80% glycerol in PBS and mounted on slides. Brightfield images were acquired with a Zeiss AxioZoom V16 Microscope.

### Identification of lncRNAs

After removing adaptors, low-quality reads as well as contaminants from the raw data in the FASTAQ files using fastp v 0.19.4 [59](default parameters), the clean reads in each library were mapped to the newest reference genome of *S*. *japonicum*(*Sj*V3) [34] using HISAT2 v 2.1.0 [60] with ‘—rna-strandness RF’ parameters. StringTie v 2.1.2 [35] was used to reconstruct transcripts with parameters of -f 0.50 -m 200 -a 10 -j 3 -c 0.1 -g 10 -fr. The transcripts were combined using StringTie-merge with reference annotation to get a set of transcripts with ‘-m 200 -F 1.0 -g 250 -f 0.05’ parameters. Then a stringent selection pipeline was developed to systemically identify lncRNAs in *S*. *japonicum*. Firstly, the transcripts shorter than 200nt were removed and the rest transcripts were examined with a merged GTF file for overlap with reference annotation using Gffcompare v0.11.2 [61]. Only transcripts with gffcompare class code ‘u’ (intergenic transcripts),’x’ (Exonic overlap with reference on the opposite strand) and’i’ (transcripts entirely within an intron) were retained. Secondly, CPC2 v0.1 [62] and FEELnc v0.1.1 [63] were used to assess the protein-coding potential. Transcripts were classified as lncRNAs or putative protein-coding mRNA using four intrinsic features through a support vector machine model in CPC2 and a random forest machine-learning algorithm in FEELnc. Thirdly, putative lncRNAs were translated in all possible six frames with transeq [64] and searched against all protein sequences in Uniport (2020_06) [65] and Pfam (v.33) [66] using Blastx 2.5.0 [67] with ‘evalue < 1e^-5^ parameters. Only transcripts that successfully passed these filters were considered reliable lncRNAs. The circos plot showing all lncRNAs on chromosomes was generated by circos software [68].

### Quantification and pattern analysis of lncRNA expression

After merging the lncRNA annotation with the *S*. *japonicum* reference gene annotation [34], we generated read counts using HTSeq 0.13.5 [69] with the parameter ‘-s yes’. Gene expression levels as CPM (counts per million) were quantified using the method TMM from the package edgeR v3.28.1 [70]. Meanwhile, RSEM v1.3.1 [71] was used to obtain transcript- and gene-level quantification with parameter ‘—strandedness reverse’. CPM value was taken as input to detect developmentally dynamic genes (Genes with a goodness-of-fit *R*^*2*^ > 0.3) using maSigPro [72]. Clustering analysis of developmentally dynamic genes was performed using the hclust function in the maSigPro package with parameter ‘k = 6’. In downstream analysis, lncRNAs with similar expression patterns in each sex were manually merged and finally classified into different representative expression patterns. Differential expression analysis of protein-coding genes and lncRNAs between female and male worms was performed using DESeq2 [73] and the sex-biased lncRNAs were defined with adjusted *P*-value < 0.05 and fold change (FC) > 2 or FC < -2.

### Weighted Gene Co-expression Network Analysis (WGCNA)

To determine the correlation between lncRNAs and mRNAs, a scale-free co-expression network was constructed based on gene profiles using WGCNA v1.6.9 [36] R package. Firstly, the Pearson correlation matrix for lncRNA-mRNA pairs was calculated and then converted into adjacency matrix a_ij_ with soft thresholding power (*β* = 12) using the “picksoftThreshold” function. Next, the adjacency matrix was converted into a Topological Overlap Matrix (TOM) that could describe the association strength between genes. The TOM was then used for the hierarchical clustering analysis. Finally, 19 gene modules were defined using the DynamicTreeCut algorithm with parameter ‘mergeCutHeight = 0.3’ and all modules were summarized by module eigengenes (ME).

To identify hub lncRNAs in each module, we calculated the Module Membership (MM) using the signed KME function from the WGCNA R package. We also calculated the Gene Significance (GS) based on the correlation between individual genes and biological traits, such as time and sex. Based on GS and MM (MM > 0.75, |GS| > 0.2), the hub lncRNAs were identified and the subnetwork of hub lncRNAs extracted from the yellow module was visualized in Cytoscape [74].

### Gene Ontology (GO) enrichment analysis

GO enrichment analysis was performed with the R package clusterProfiler [75]. The *P*-values were corrected for multiple hypothesis testing with the Benjamini–Hochberg false-discovery rate procedure.

### Statistical analysis

Statistical analyses were performed with GraphPad Prism (version 8.0). *P*-value or adjusted *P*-value <0.05 was considered statistically significant.

### Data sharing statement

The raw sequencing data reported in this paper have been deposited in the NCBI Sequence Read Archive (SRA) database under the accession number PRJNA992996 and can be accessed via link https://www.ncbi.nlm.nih.gov/bioproject/PRJNA992996. The *S*.*japonicum* version 3 genome sequences utilized in our study [34], along with the annotations for the identified long non-coding RNAs and protein-coding gene annotations, are available for download at the following link: https://doi.org/10.5061/dryad.x95x69prn [76].

## Supporting information

S1 Fig Venn diagram representing the lncRNAs identified by four methods. (TIF)

S2 Fig Heatmap of pearson correlation coefficient for 21 samples based on the lncRNA expression level. The samples were grouped by hierarchical clustering.
(TIF)

S3 Fig Developmental dynamics of lncRNAs and their expression pattern in *S*. *japonicum*. (A) The number of genes expressing lncRNAs (Transcripts Per Kilobase Million, TPM ≥ 1) detected at different developmental time points in male and female worms (mean ± sem, n = 3). (B) Number of developmentally dynamic and non-dynamic lncRNAs and mRNAs identified in male and female worms. Genes with a goodness-of-fit (R^2^) > 0.3 were classified as developmentally dynamic. (C) Average expression levels of developmental dynamic lncRNAs and mRNA genes. (D)Unmodified expression patterns clusters of developmental dynamic lncRNAs during reproductive development in females (top) and males (down). The developmentally dynamic genes will be clustered into 6 different expression patterns by hierarchical clustering using “hclust” function in maSigPro package, and then similar expression patterns of lncRNAs were manually merged for downstream analysis (Cluster2 and Cluster3 were merged to one in female; Cluster 1–4 were into merged one in male). Lines estimated through LOESS regression; 95% confidence interval shown in grey.
(TIF)

S4 Fig Differential expression analysis and identification of sex-biased lncRNAs. (A) Volcano plots of sex-biased lncRNAs at three time points. Left: 18 dpi, middle: 22 dpi, right: 26 dpi The sex-biased lncRNAs were obtained by DEseq2 (Cut-off: |Log_2_FC| ≥1, adjusted *P*-value < 0.05). (B) Hierarchical clustering heat map representing the sex-biased lncRNAs expression level in all samples.
(TIF)

S5 Fig Eigengene bar plots of lncRNAs-mRNA co-expression modules. The color of each bar plot is consistent with the corresponding module name. The Y-axis represents the normalized expression level of the eigengene in each sample, and the X-axis represents the sequencing samples arranged by time and gender. The number of lncRNAs and mRNA genes in each module is marked above the histogram.
(TIF)

S6 Fig Analysis of egg-laying and LncRNA expression following RNA interference. (A) the number of eggs per worm pair after the treatment of GFP or LncRNA dsRNA for 8 days *in vitro*. No statistically significant differences were observed across the seven LncRNA interference groups. (B) Relative mRNA expression levels of the seven LncRNA in both male and female worms post-RNAi, as determined by qPCR (mean ± SE, n = 3).
(TIF)

S7 Fig NAT-lncRNAs co-expression with cognate sense genes. (A) Scatter plots of expression changes of *LNC3385* and (B) *Sjc_0009416* in female (left) and male (right) at four-time points during reproductive development, with LOESS regression lines added and 95% confidence intervals shown in gray. (C) Scatter plot showing the relationship of expression levels between *LNC3385* (on the x-axis) and its cognate sense genes *Sjc_0009416* (on the y-axis). Each point represents an individual data point. The correlation coefficient (R) is -0.86 and the *P*-value is equal to 4.5e^-7^. (D) Whole-mount *in situ* hybridization of the *LNC3385* gene at different time points during reproductive development in male and female worms, presented in the upper images, with a negative control displayed at the bottom. Each image illustrates two segments of the female worm, displaying the entire organism. (E) qPCR results of *RERE* and *LNC3385* expression levels in *GFP* RNAi and *RERE* RNAi conditions in both female and male samples. The y-axis represents the relative gene expression levels of *RERE* and *LNC3385*, while the x-axis denotes the different RNAi conditions and sexes. Red bars represent *GFP* RNAi samples and grey bars represent *RERE* RNAi samples. * *P*-value < 0.05; ** *P*-value < 0.01; *** *P*-value < 0.005; **** *P*-value < 0.001; n.s. not statistically significant.
(TIF)

S8 Fig Representative images of carmalum staining for the reproductive organs of parasites from the LNC3385 and GFP RNAi group under an optical microscope. (TIF)

S9 Fig Altered mouse liver pathology after *in vivo* knock-down of *LNC3385*. (A) Liver morphology in control vs. *LNC3385* RNAi-treated mice. (B) HE (hematoxylin and eosin) and (C) Masson’s trichrome staining of livers from mice harboring GFP or *LNC3385* RNAi parasites. (D) The number of eggs per pair after the treatment of GFP or *LNC3385* dsRNA *in vivo*. (E) Bar plot depicts the average granuloma area surrounding a single egg in *LNC3385* dsRNA-treated versus GFP control group.
(TIF)

S10 Fig Hierarchical clustering heatmap representing differential gene expression following *LNC3385* RNAi. The heatmap depicts the changes in gene expression levels after the RNA interference (RNAi) of *LNC3385*. Each row represents a gene, and each column represents a sample. The color scale on the right represents the relative expression level of each gene, with red indicating upregulation and blue indicating downregulation.
(TIF)

S11 Fig ATAC-seq data quality control and evaluation of the correlation between Samples. (A) Distribution of ATAC-seq fragment size in each sample. (B) A heatmap detailing chromatin accessibility around the transcript start site (TSS), with a meta plot displayed above the heatmap to provide a summary view of TSS accessibility across all samples. (C) A Principal Component Analysis (PCA) plot of all samples to highlight the variability and similarities in chromatin accessibility patterns between GFP and *LNC3385* RNAi samples. (D) Heatmap clustering across all 6 samples ATAC-seq profiles.
(TIF)

S12 Fig Genomic localization and accessibility of *LNC3385* locus from ATAC-seq Data. (A) A genome track plot of the *LNC3385* locus displaying the ATAC-seq signals. ATAC-seq peaks are indicated with corresponding color-coded bars below the tracks. A genome scale bar with the *RERE* and *LNC3385* loci is shown below. (B) A pie chart illustrating the proportion of all long non-coding RNAs that overlap with chromatin accessible regions (ACRs).
(TIF)

S13 Fig Characterization of ATAC peaks and gene expression following *LNC3385* RNAi. (A) Volcano plot of differentially accessible chromatin regions (Cut-off: |Log_2_FC| ≥ 0.5, adjusted *P*-value < 0.05). (B) The Venn diagram depicts the intersection between neighboring genes of significantly downregulated peaks in ATAC-seq data (ATAC_NeighboringDownGenes) and downregulated genes within the co-expressed network regulated by *LNC3385* detected in RNA-seq analysis (CoExpNet_LNC3385_DownRegGenes, fold change < -0.15 and *P*-value < 0.05). (C) Genome browser view of ATAC-seq signal surrounding the *Sjc_0008020* gene, with ATAC-seq peaks represented by color-coded bars beneath the tracks, providing a visual representation of the genomic locations of chromatin accessibility changes.
(TIF)

S1 Table A summary of *S*.*japonicum* strand-specific RNA-seq data. (XLSX)

S2 Table Detailed annotation and comparison of identified lncRNAs. (XLSX)

S3 Table Sex-biased expression of lncRNAs in *S*. *japonicum*. (XLSX)

S4 Table WGCNA co-expression modules of lncRNAs and mRNAs in *S*. *japonicum*. (XLSX)

S5 Table GO terms enrichment for the mRNAs from the lncRNA-mRNA co-expression modules. (XLSX)

S6 Table A subnetwork of hub-lncRNAs and their co-expression mRNAs within yellow module. (XLSX)

S7 Table Differential expressed LncRNAs and protein-coding genes after *LNC3385* RNAi. (XLSX)

S8 Table A summary of *S*.*japonicum* ATAC-seq data after *LNC3385* RNAi. (XLSX)

S9 Table Differential accessibility ATAC-seq peaks after *LNC3385* RNAi. (XLSX)

S10 Table Changes in chromatin accessibility of predicted target genes after *LNC3385* RNAi. (XLSX)

S1 File Multi-parameter BLASTN output results for comparative analysis of identified lncRNAs by Maciel *et al*. (XLSX)
