# Supplementary figures and images for: Dynamic profiles of lncRNAs reveal a functional natural antisense RNA that regulates the development of Schistosoma japonicum
Source: PLoS Pathog. 2024 Jan 29;20(1):e1011949. doi: 10.1371/journal.ppat.1011949 (PMC10878521; doi:10.1371/journal.ppat.1011949)

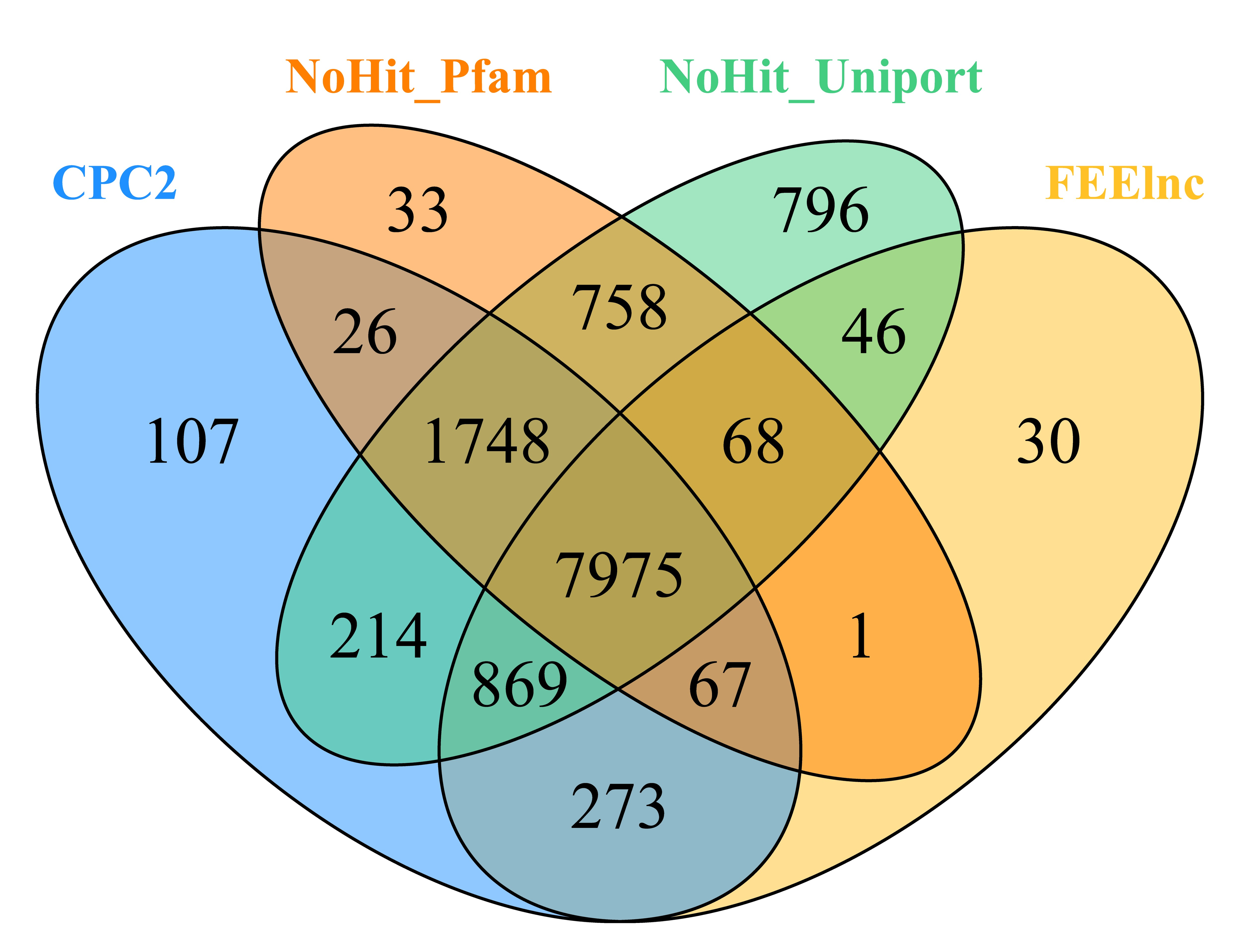

Supplement: S1 Fig — (TIF) [file ppat.1011949.s001.tif]

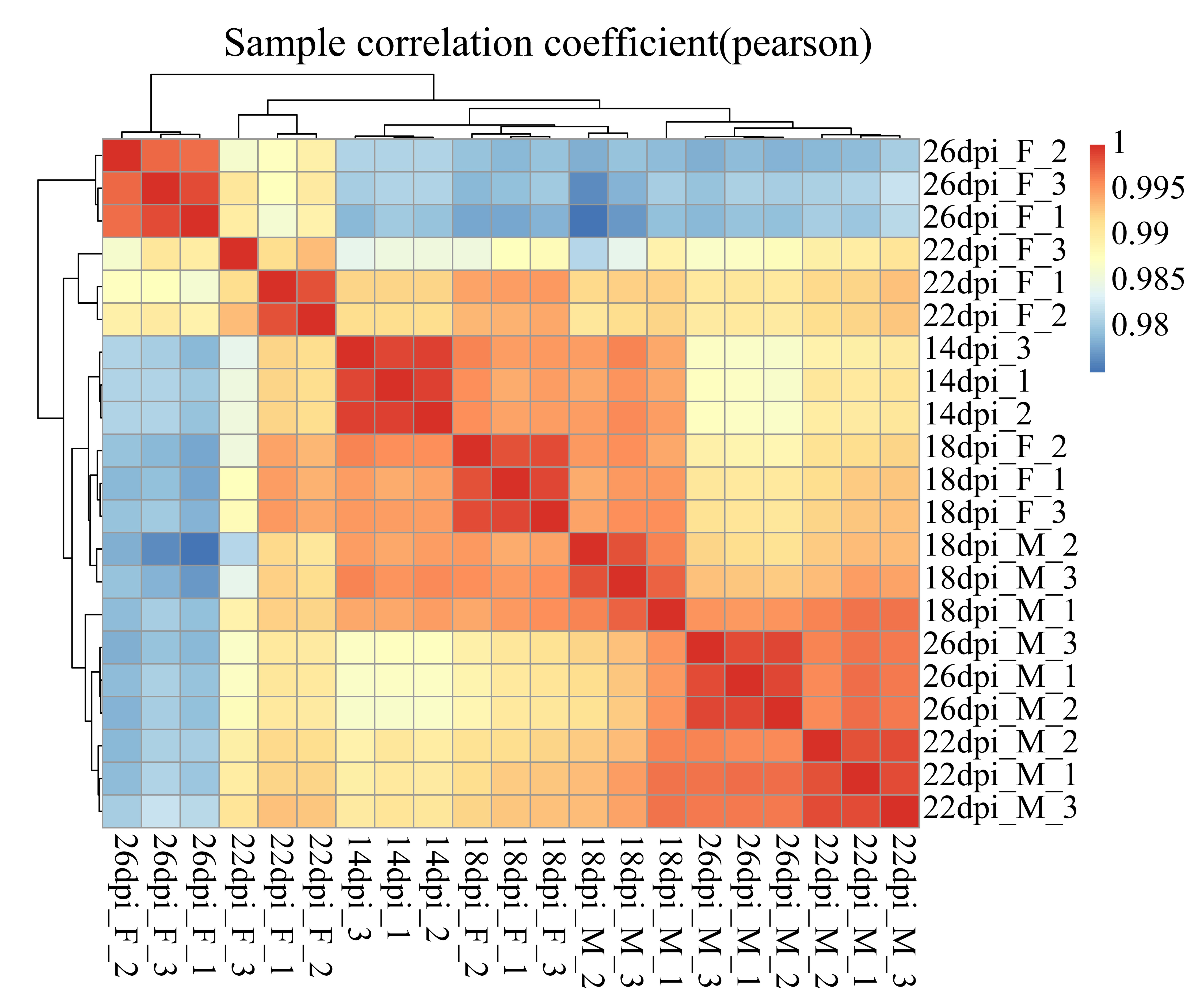

Supplement: S2 Fig — The samples were grouped by hierarchical clustering. (TIF) [file ppat.1011949.s002.tif]

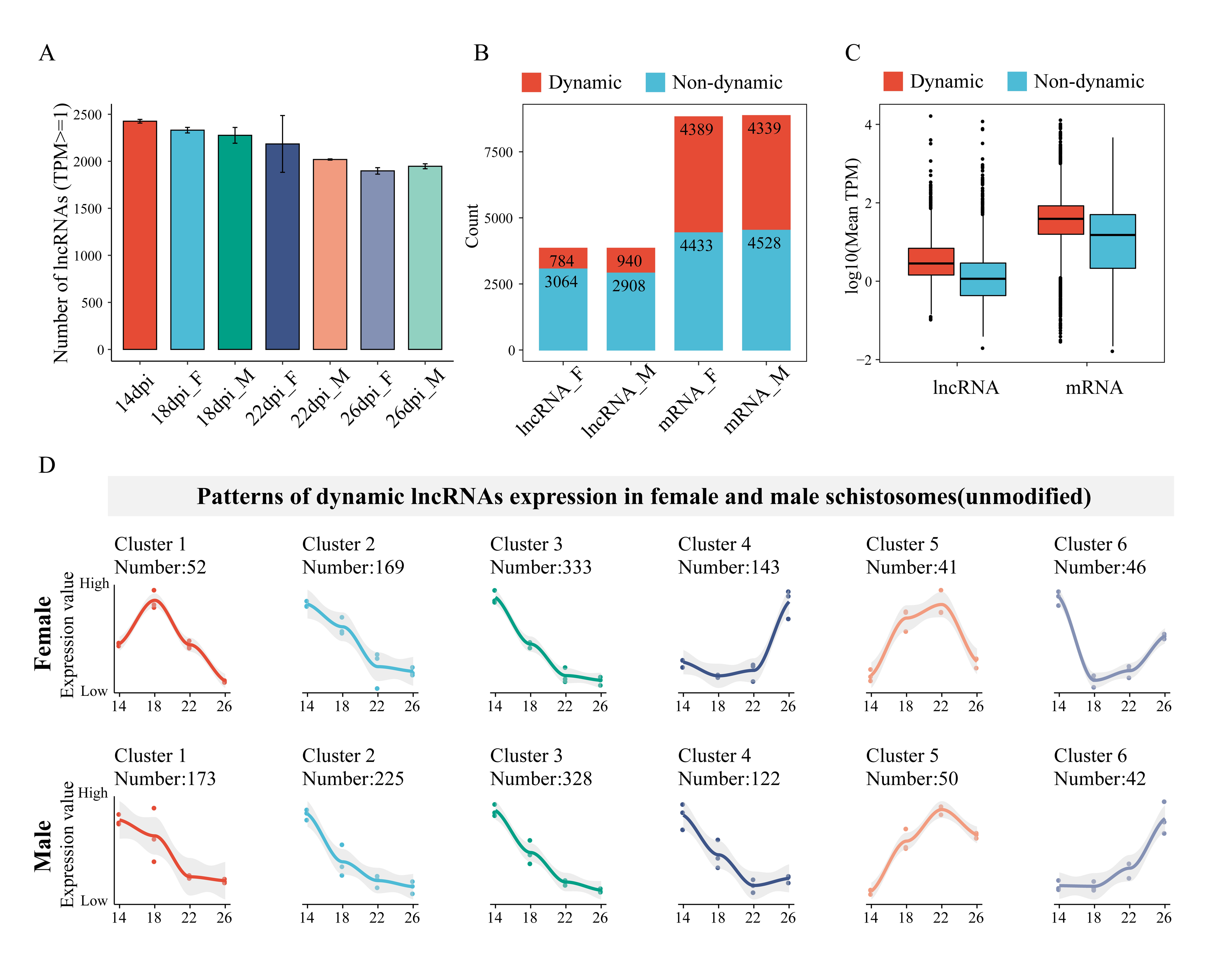

Supplement: S3 Fig — (A) The number of genes expressing lncRNAs (Transcripts Per Kilobase Million, TPM ≥ 1) detected at different developmental time points in male and female worms (mean ± sem, n = 3). (B) Number of developmentally dynamic and non-dynamic lncRNAs and mRNAs identified in male and female worms. Genes with a goodness-of-fit (R2) > 0.3 were classified as developmentally dynamic. (C) Average expression levels of developmental dynamic lncRNAs and mRNA genes. (D)Unmodified expression patterns clusters of developmental dynamic lncRNAs during reproductive development in females (top) and males (down). The developmentally dynamic genes will be clustered into 6 different expression patterns by hierarchical clustering using “hclust” function in maSigPro package, and then similar expression patterns of lncRNAs were manually merged for downstream analysis (Cluster2 and Cluster3 were merged to one in female; Cluster 1–4 were into merged one in male). Lines estimated through LOESS regression; 95% confidence interval shown in grey. (TIF) [file ppat.1011949.s003.tif]

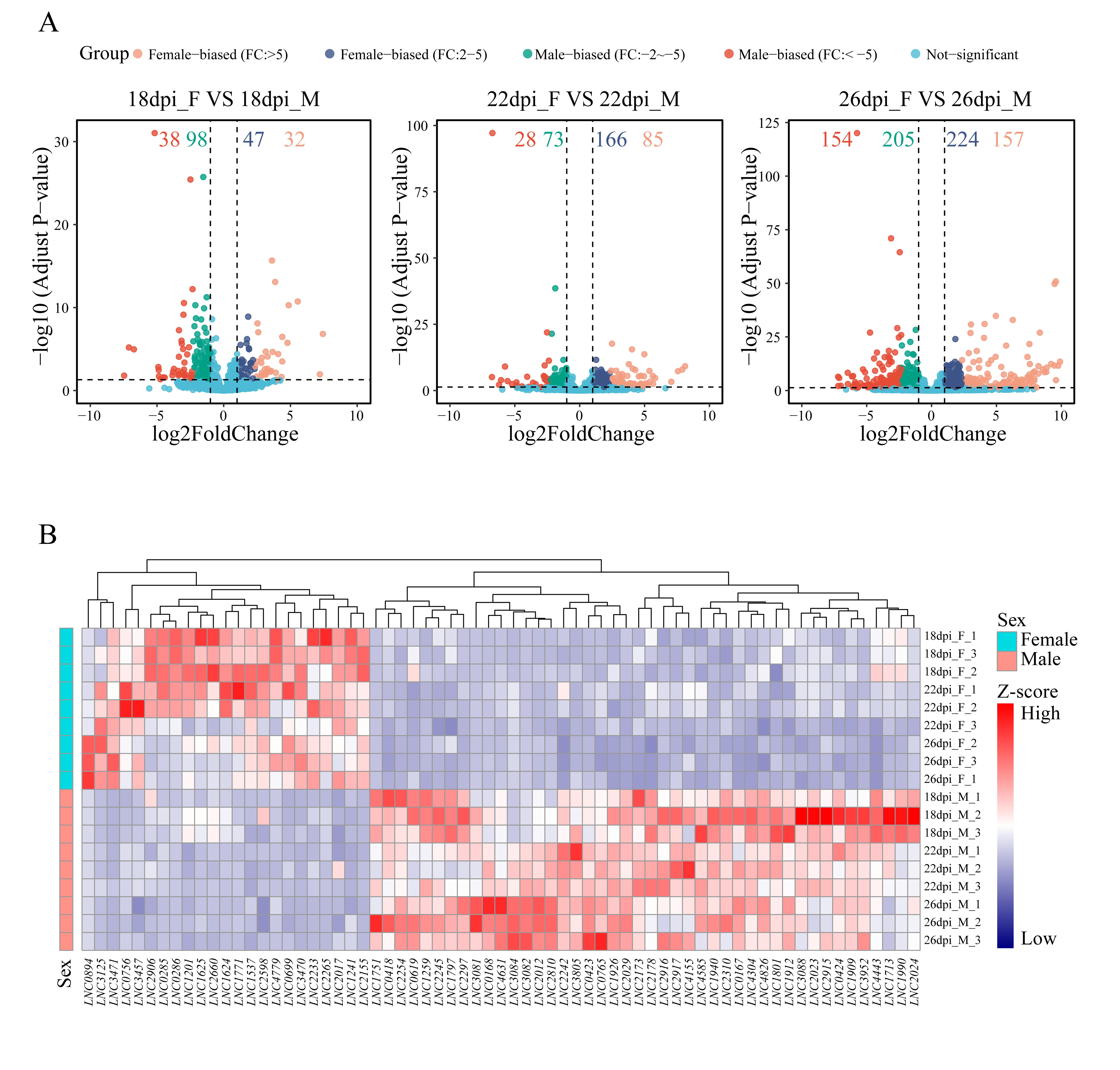

Supplement: S4 Fig — (A) Volcano plots of sex-biased lncRNAs at three time points. Left: 18 dpi, middle: 22 dpi, right: 26 dpi The sex-biased lncRNAs were obtained by DEseq2 (Cut-off: |Log2FC| ≥1, adjusted P-value < 0.05). (B) Hierarchical clustering heat map representing the sex-biased lncRNAs expression level in all samples. (TIF) [file ppat.1011949.s004.tif]

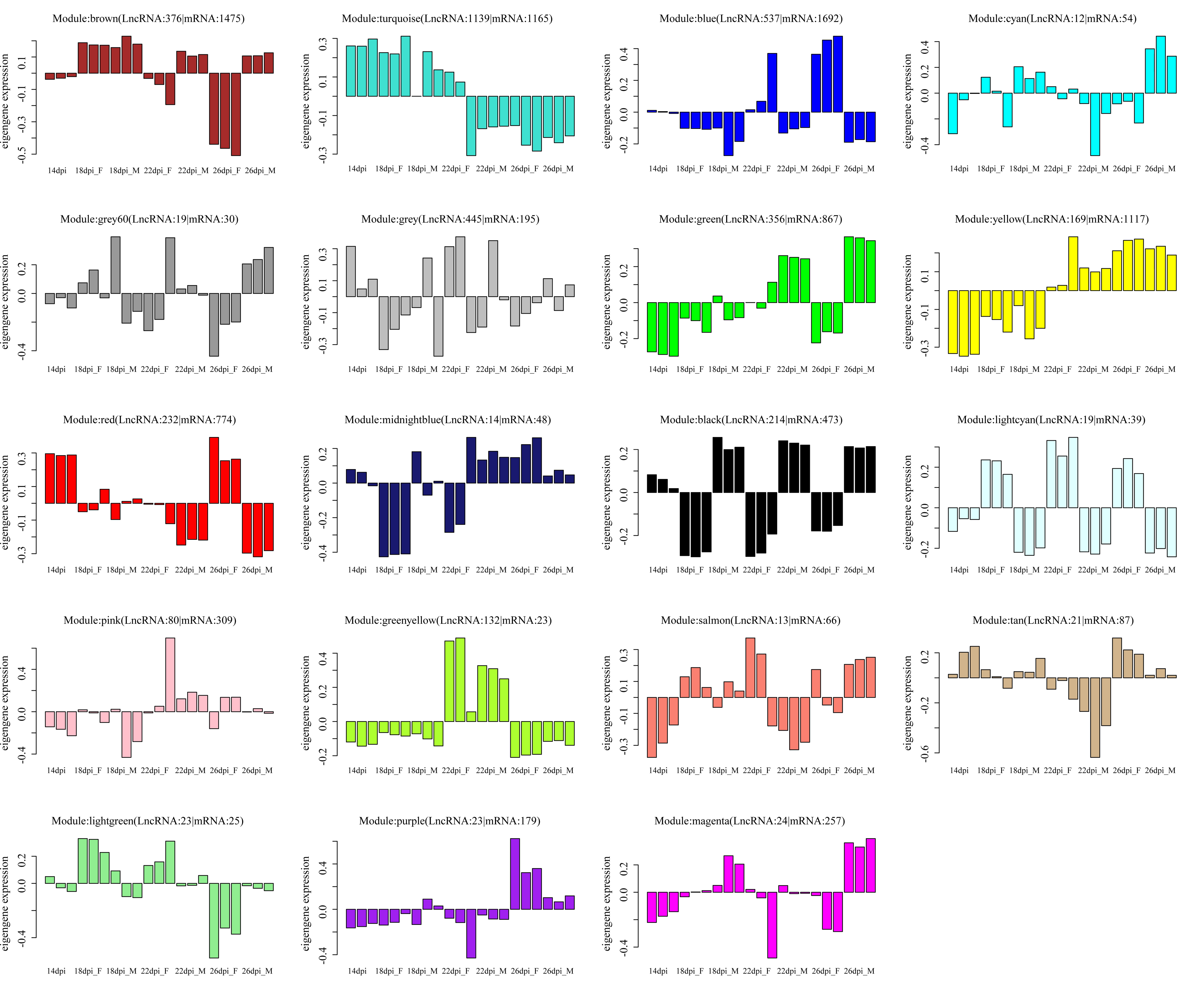

Supplement: S5 Fig — The color of each bar plot is consistent with the corresponding module name. The Y-axis represents the normalized expression level of the eigengene in each sample, and the X-axis represents the sequencing samples arranged by time and gender. The number of lncRNAs and mRNA genes in each module is marked above the histogram. (TIF) [file ppat.1011949.s005.tif]

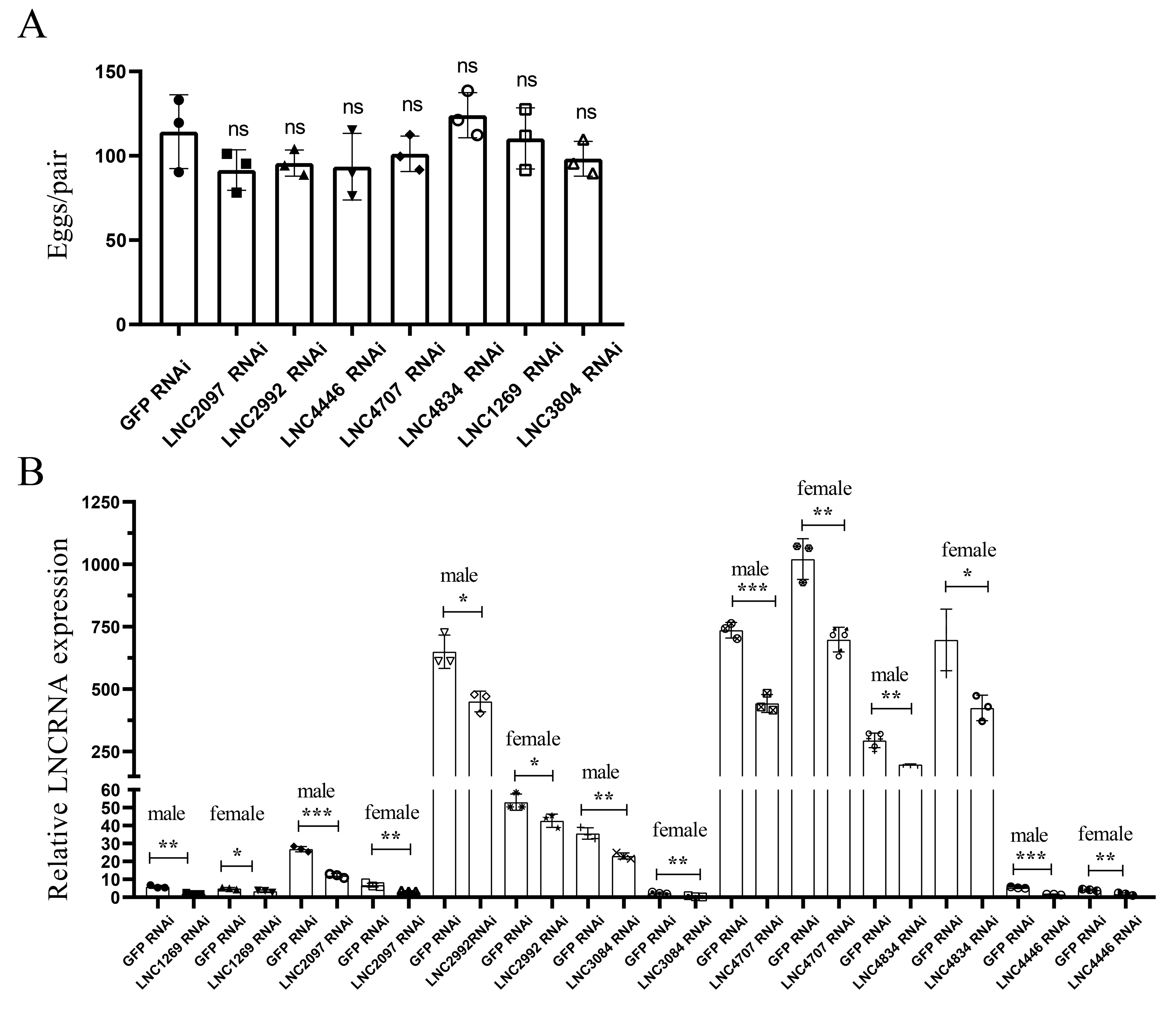

Supplement: S6 Fig — (A) the number of eggs per worm pair after the treatment of GFP or LncRNA dsRNA for 8 days in vitro. No statistically significant differences were observed across the seven LncRNA interference groups. (B) Relative mRNA expression levels of the seven LncRNA in both male and female worms post-RNAi, as determined by qPCR (mean ± SE, n = 3). (TIF) [file ppat.1011949.s006.tif]

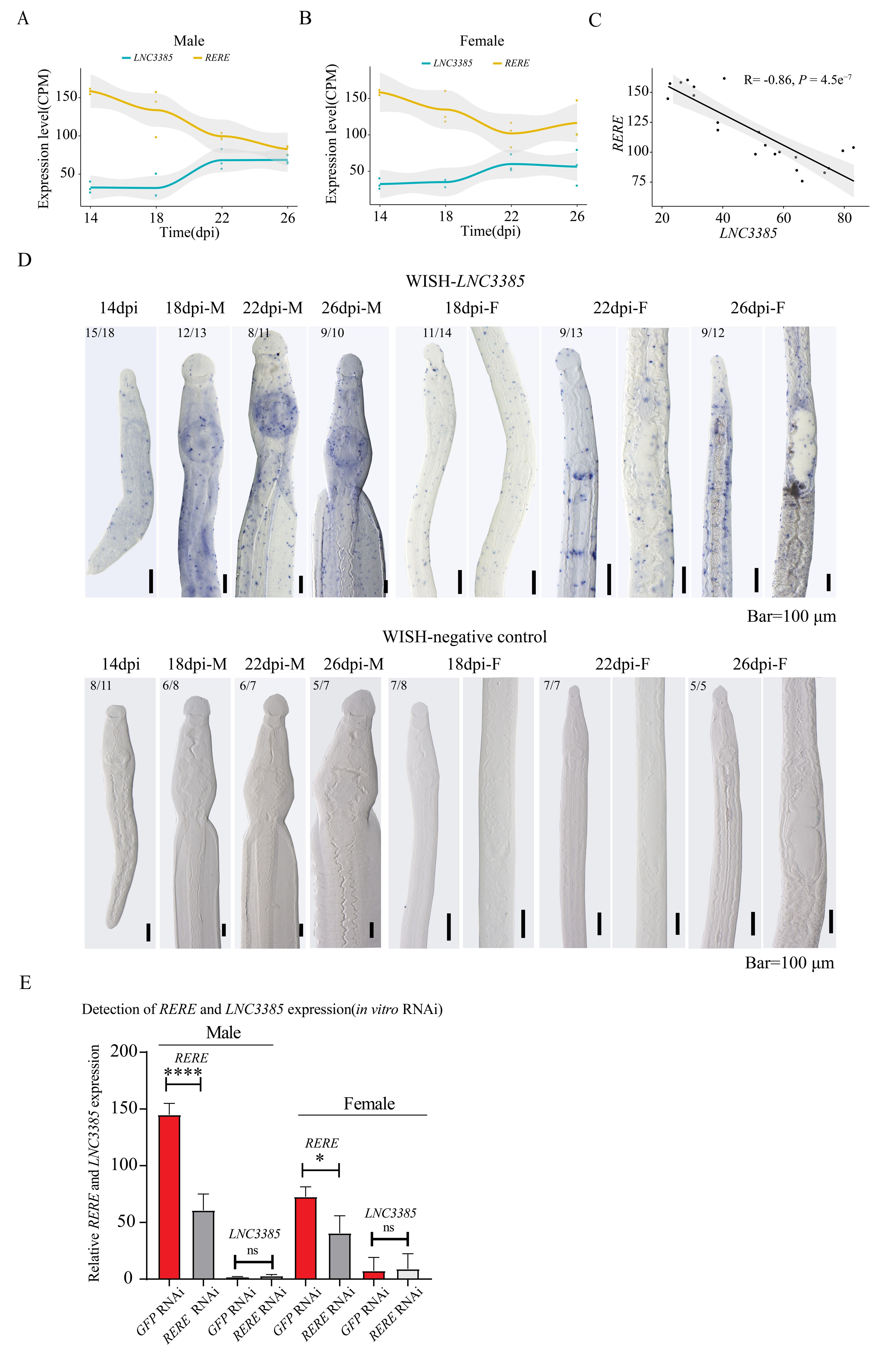

Supplement: S7 Fig — (A) Scatter plots of expression changes of LNC3385 and (B) Sjc_0009416 in female (left) and male (right) at four-time points during reproductive development, with LOESS regression lines added and 95% confidence intervals shown in gray. (C) Scatter plot showing the relationship of expression levels between LNC3385 (on the x-axis) and its cognate sense genes Sjc_0009416 (on the y-axis). Each point represents an individual data point. The correlation coefficient (R) is -0.86 and the P-value is equal to 4.5e-7. (D) Whole-mount in situ hybridization of the LNC3385 gene at different time points during reproductive development in male and female worms, presented in the upper images, with a negative control displayed at the bottom. Each image illustrates two segments of the female worm, displaying the entire organism. (E) qPCR results of RERE and LNC3385 expression levels in GFP RNAi and RERE RNAi conditions in both female and male samples. The y-axis represents the relative gene expression levels of RERE and LNC3385, while the x-axis denotes the different RNAi conditions and sexes. Red bars represent GFP RNAi samples and grey bars represent RERE RNAi samples. * P-value < 0.05; ** P-value < 0.01; *** P-value < 0.005; **** P-value < 0.001; n.s. not statistically significant. (TIF) [file ppat.1011949.s007.tif]

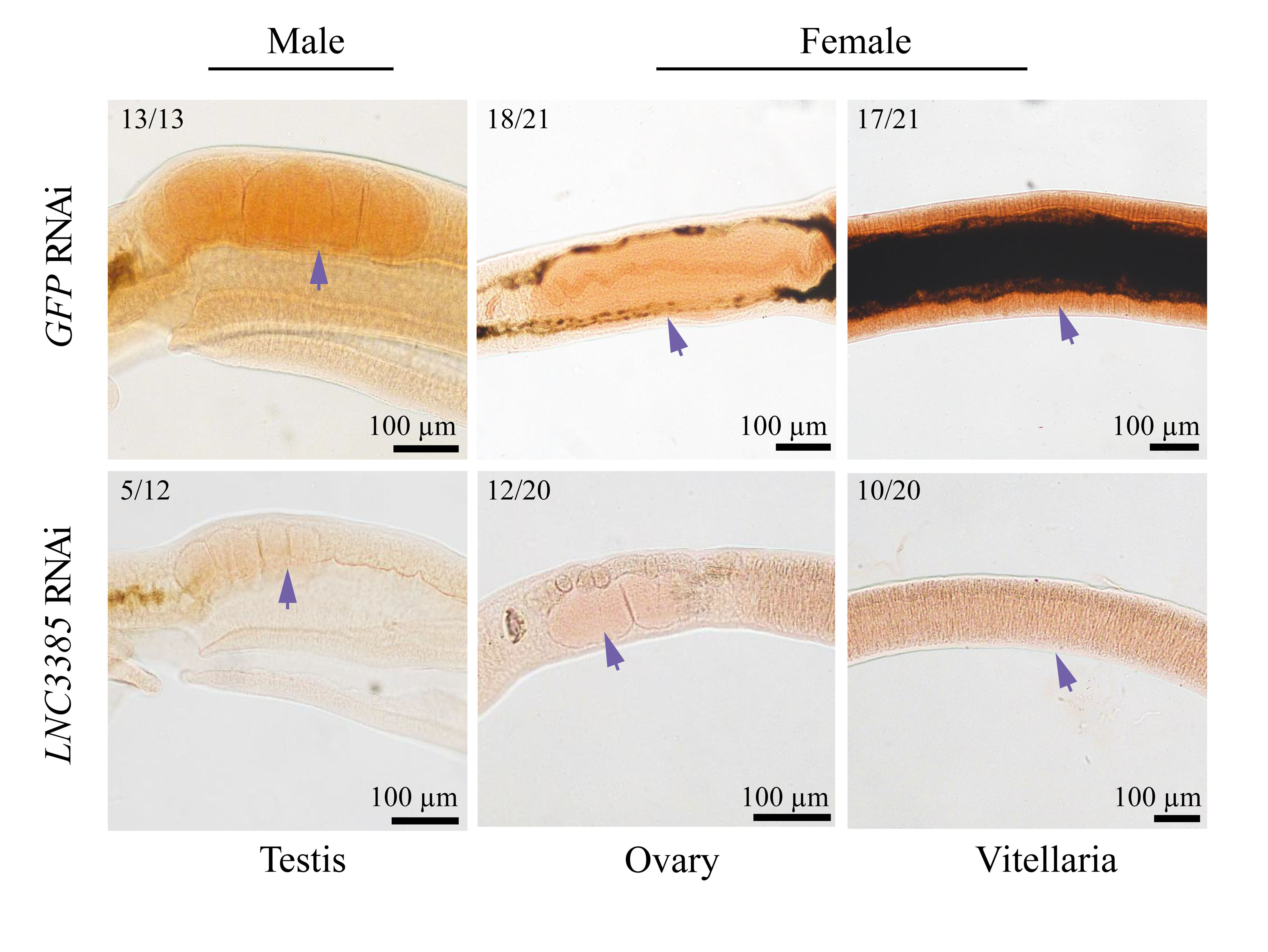

Supplement: S8 Fig — (TIF) [file ppat.1011949.s008.tif]

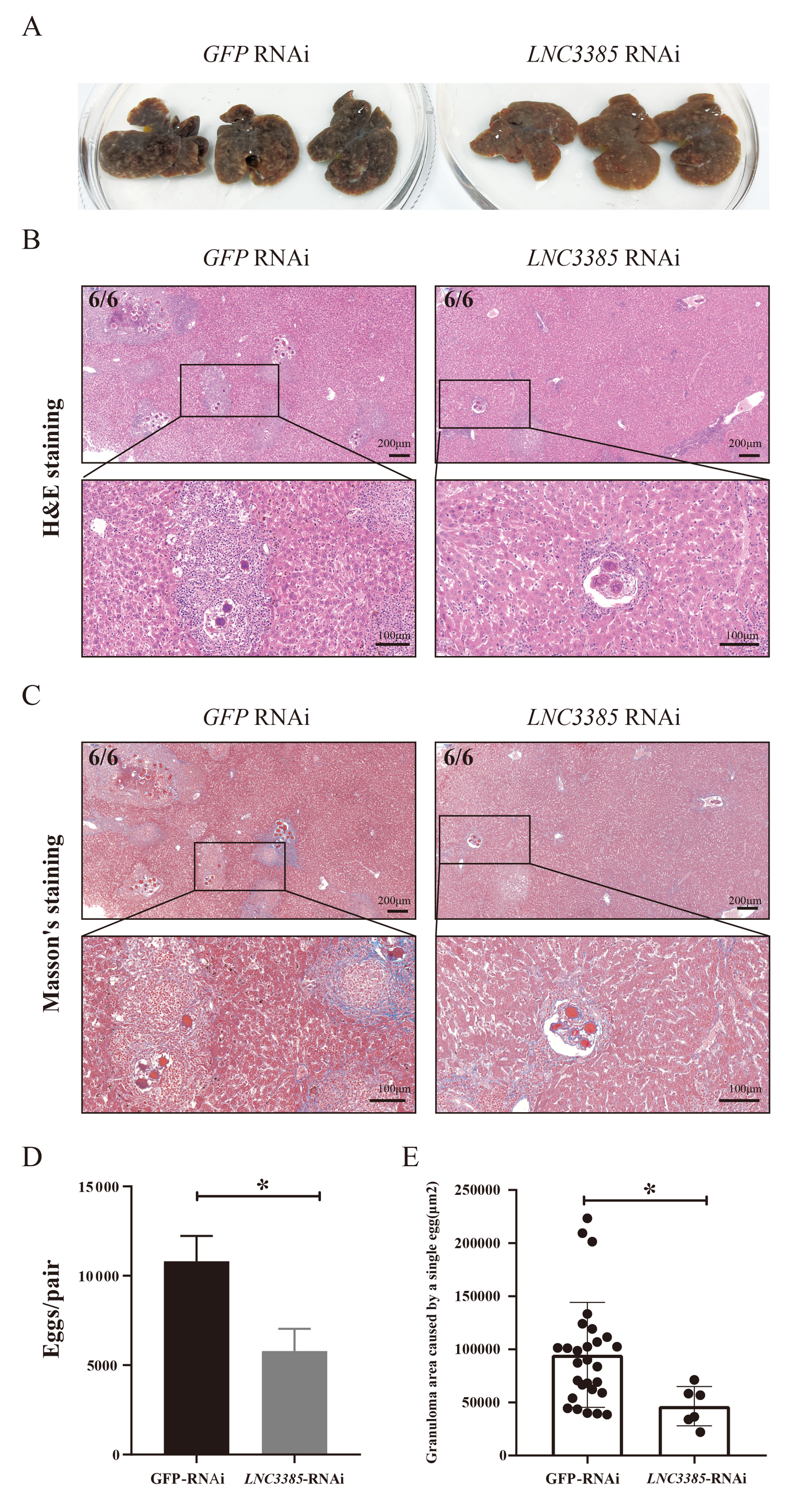

Supplement: S9 Fig — (A) Liver morphology in control vs. LNC3385 RNAi-treated mice. (B) HE (hematoxylin and eosin) and (C) Masson’s trichrome staining of livers from mice harboring GFP or LNC3385 RNAi parasites. (D) The number of eggs per pair after the treatment of GFP or LNC3385 dsRNA in vivo. (E) Bar plot depicts the average granuloma area surrounding a single egg in LNC3385 dsRNA-treated versus GFP control group. (TIF) [file ppat.1011949.s009.tif]

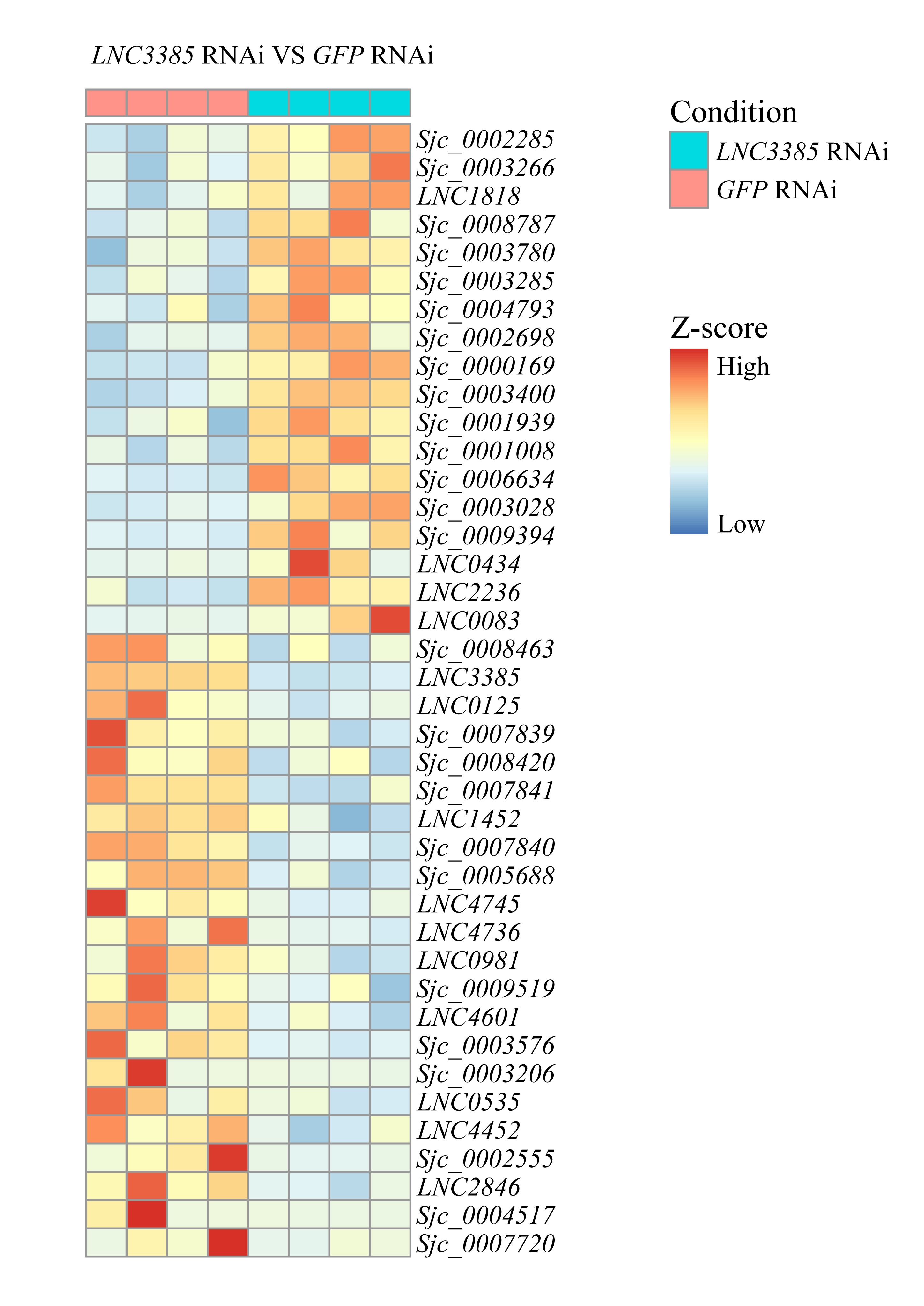

Supplement: S10 Fig — The heatmap depicts the changes in gene expression levels after the RNA interference (RNAi) of LNC3385. Each row represents a gene, and each column represents a sample. The color scale on the right represents the relative expression level of each gene, with red indicating upregulation and blue indicating downregulation. (TIF) [file ppat.1011949.s010.tif]

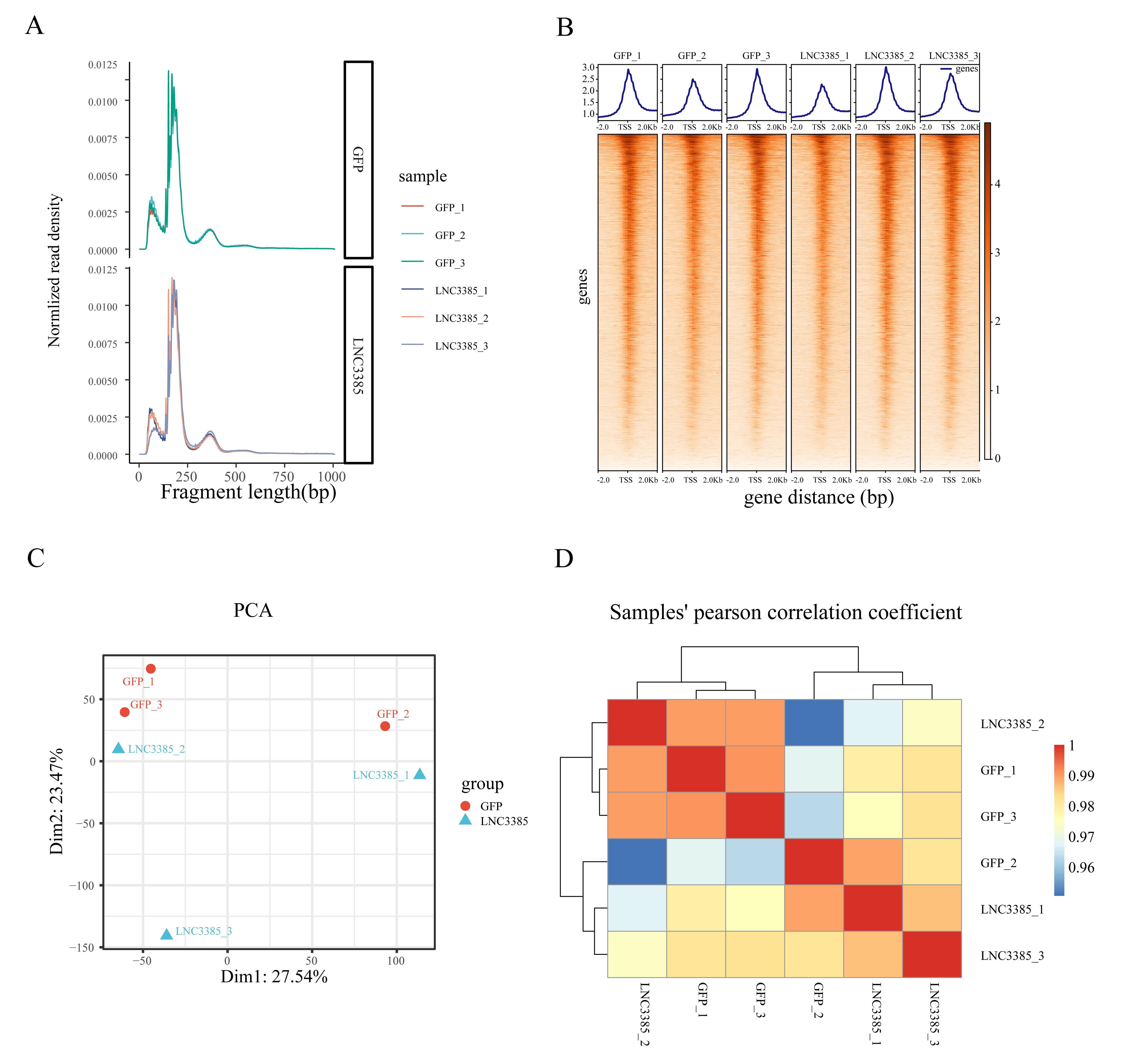

Supplement: S11 Fig — (A) Distribution of ATAC-seq fragment size in each sample. (B) A heatmap detailing chromatin accessibility around the transcript start site (TSS), with a meta plot displayed above the heatmap to provide a summary view of TSS accessibility across all samples. (C) A Principal Component Analysis (PCA) plot of all samples to highlight the variability and similarities in chromatin accessibility patterns between GFP and LNC3385 RNAi samples. (D) Heatmap clustering across all 6 samples ATAC-seq profiles. (TIF) [file ppat.1011949.s011.tif]

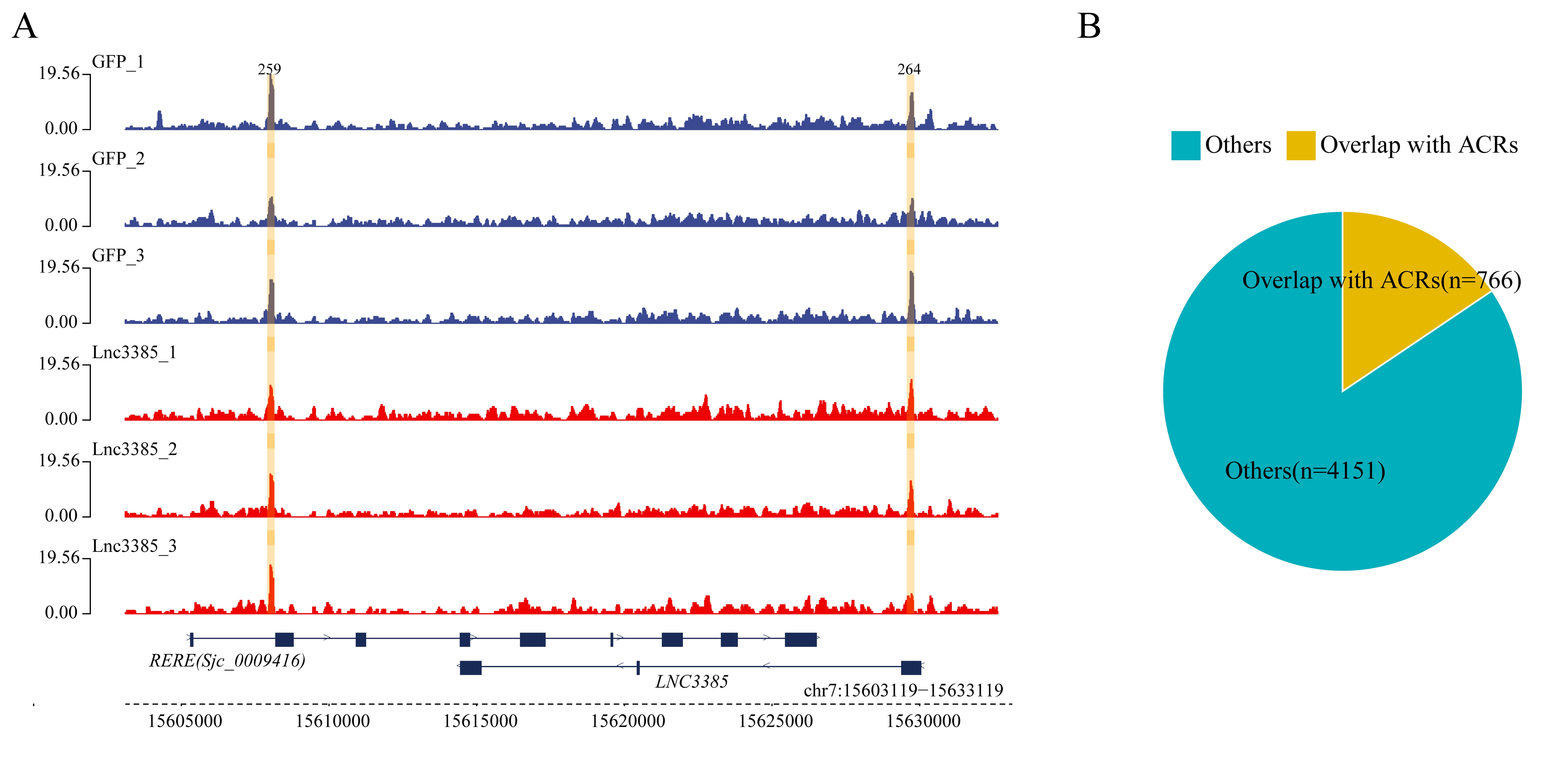

Supplement: S12 Fig — (A) A genome track plot of the LNC3385 locus displaying the ATAC-seq signals. ATAC-seq peaks are indicated with corresponding color-coded bars below the tracks. A genome scale bar with the RERE and LNC3385 loci is shown below. (B) A pie chart illustrating the proportion of all long non-coding RNAs that overlap with chromatin accessible regions (ACRs). (TIF) [file ppat.1011949.s012.tif]

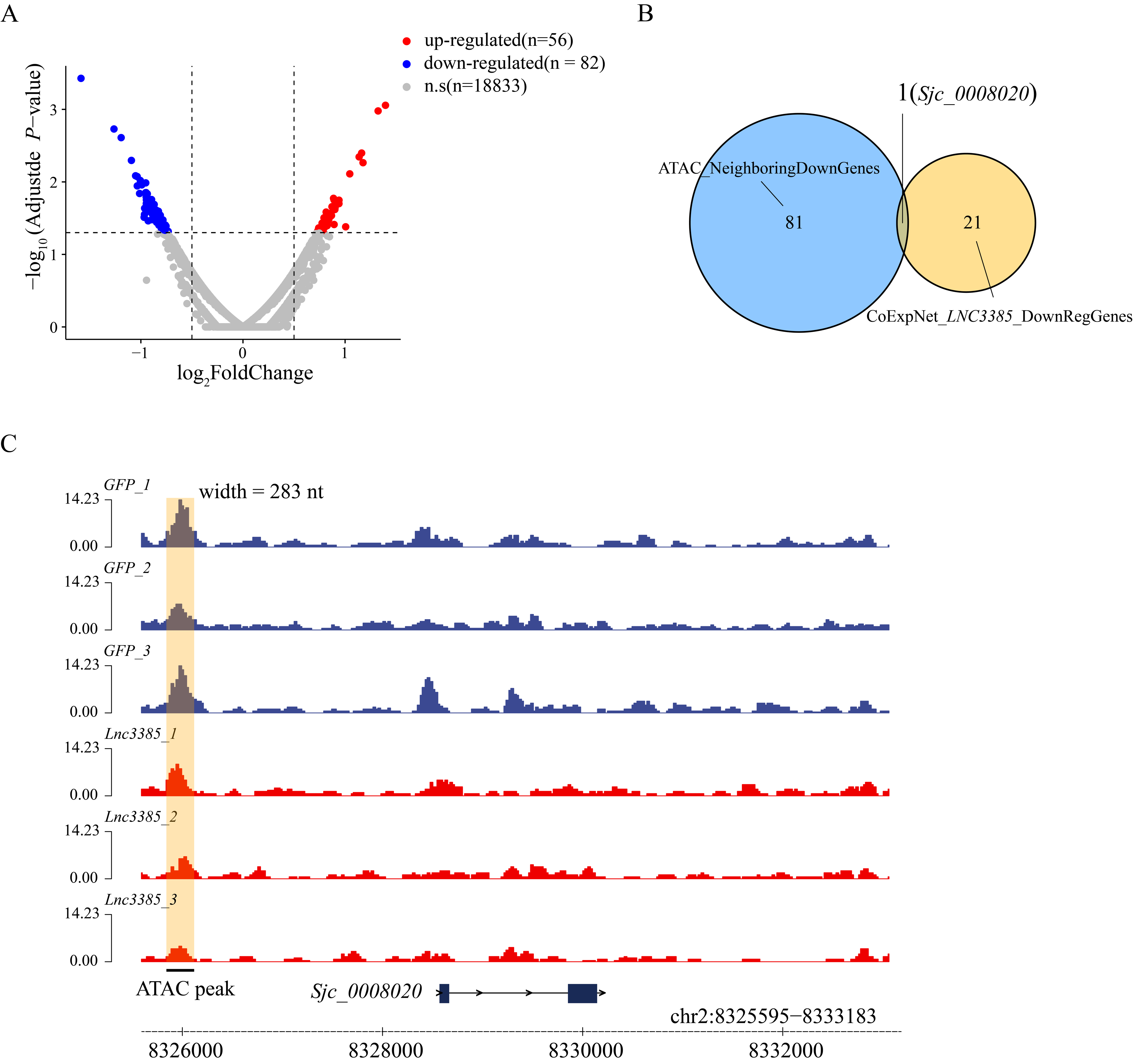

Supplement: S13 Fig — (A) Volcano plot of differentially accessible chromatin regions (Cut-off: |Log2FC| ≥ 0.5, adjusted P-value < 0.05). (B) The Venn diagram depicts the intersection between neighboring genes of significantly downregulated peaks in ATAC-seq data (ATAC_NeighboringDownGenes) and downregulated genes within the co-expressed network regulated by LNC3385 detected in RNA-seq analysis (CoExpNet_LNC3385_DownRegGenes, fold change < -0.15 and P-value < 0.05). (C) Genome browser view of ATAC-seq signal surrounding the Sjc_0008020 gene, with ATAC-seq peaks represented by color-coded bars beneath the tracks, providing a visual representation of the genomic locations of chromatin accessibility changes. (TIF) [file ppat.1011949.s013.tif]
